# Development and Validation of a Dynamic Real-Time Risk Prediction Model for Intensive Care Units Patients Based on Longitudinal Irregular Data: Multicenter Retrospective Study

**DOI:** 10.2196/69293

**Published:** 2025-04-23

**Authors:** Zhuo Zheng, Jiawei Luo, Yingchao Zhu, Lei Du, Lan Lan, Xiaobo Zhou, Xiaoyan Yang, Shixin Huang

**Affiliations:** 1 Department of Anesthesiology West China Hospital of Sichuan University Chengdu China; 2 West China Biomedical Big Data Center West China Hospital of Sichuan University Chengdu China; 3 Information Management and Data Center Beijing Tiantan Hospital Beijing China; 4 Center for Computational Systems Medicine McWilliams School of Biomedical Informatics The University of Texas Health Science Center at Houston Houston, TX United States; 5 Department of Scientific Research The People’s Hospital of Yubei District of Chongqing City Chongqing China

**Keywords:** intensive care units, machine learning, in-hospital mortality, continuous prediction, model interpretability

## Abstract

**Background:**

Timely and accurate prediction of short-term mortality is critical in intensive care units (ICUs), where patients’ conditions change rapidly. Traditional scoring systems, such as the Simplified Acute Physiology Score and Acute Physiology and Chronic Health Evaluation, rely on static variables collected within the first 24 hours of admission and do not account for continuously evolving clinical states. These systems lack real-time adaptability, interpretability, and generalizability. With the increasing availability of high-frequency electronic medical record (EMR) data, machine learning (ML) approaches have emerged as powerful tools to model complex temporal patterns and support dynamic clinical decision-making. However, existing models are often limited by their inability to handle irregular sampling and missing values, and many lack rigorous external validation across institutions.

**Objective:**

We aimed to develop a real-time, interpretable risk prediction model that continuously assesses ICU patient mortality using irregular, longitudinal EMR data, with improved performance and generalizability over traditional static scoring systems.

**Methods:**

A time-aware bidirectional attention-based long short-term memory (TBAL) model was developed using EMR data from the MIMIC-IV (Medical Information Mart for Intensive Care) and eICU Collaborative Research Database (eICU-CRD) databases, comprising 176,344 ICU stays. The model incorporated dynamic variables, including vital signs, laboratory results, and medication data, updated hourly, to perform static and continuous mortality risk assessments. External cross-validation and subgroup sensitivity analyses were conducted to evaluate robustness and fairness. Model performance was assessed using the area under the receiver operating characteristic curve (AUROC), area under the precision-recall curve (AUPRC), accuracy, and *F*_1_-score. Interpretability was enhanced using integrated gradients to identify key predictors.

**Results:**

For the static 12-hour to 1-day mortality prediction task, the TBAL model achieved AUROCs of 95.9 (95% CI 94.2-97.5) and 93.3 (95% CI 91.5-95.3) and AUPRCs of 48.5 and 21.6 in MIMIC-IV and eICU-CRD, respectively. Accuracy and *F*_1_-scores reached 94.1 and 46.7 in MIMIC-IV and 92.2 and 28.1 in eICU-CRD. In dynamic prediction tasks, AUROCs reached 93.6 (95% CI 93.2-93.9) and 91.9 (95% CI 91.6-92.1), with AUPRCs of 41.3 and 50, respectively. The model maintained high recall for positive cases (82.6% and 79.1% in MIMIC-IV and eICU-CRD). Cross-database validation yielded AUROCs of 81.3 and 76.1, confirming generalizability. Subgroup analysis showed stable performance across age, sex, and severity strata, with top predictors including lactate, vasopressor use, and Glasgow Coma Scale score.

**Conclusions:**

The TBAL model offers a robust, interpretable, and generalizable solution for dynamic real-time mortality risk prediction in ICU patients. Its ability to adapt to irregular temporal patterns and to provide hourly updated predictions positions it as a promising decision-support tool. Future work should validate its utility in prospective clinical trials and investigate its integration into real-world ICU workflows to enhance patient outcomes.

## Introduction

The intensive care unit (ICU) is a critical environment where timely and accurate decisions can significantly impact patient outcomes. Predicting the risk of adverse events, especially mortality, is essential for guiding clinical management [1,2]. ICUs provide continuous monitoring, advanced treatment, and diagnostic technologies. However, ICU clinicians face overwhelming amounts of patient data stored in electronic Patient Data Management Systems. It is becoming increasingly difficult to identify the most important information for care decisions [3]. The human ability to process such large volumes of information is limited, leading to risks such as data overload, inattentional blindness, and task fixation. These factors increase the likelihood that clinicians may fail to recognize, interpret, or act on relevant information [4,5]. Traditional prognostic models, such as the Simplified Acute Physiology Score (SAPS) and the Acute Physiology and Chronic Health Evaluation (APACHE), have been widely used to assess disease severity and predict mortality in ICU patients [6-10]. However, these models have limitations, including low accuracy, reliance on static data, and dependence on information from the first day of ICU admission. They fail to account for the dynamically changing clinical state of patients during their ICU stay. Additionally, the lack of personalized prediction tools often forces clinicians to rely on subjective judgment, which can lead to biased decisions and missed opportunities for timely intervention [11-13].

Recent advances in machine learning (ML) offer a promising solution to these challenges [14-16]. ML algorithms can process large, heterogeneous, high-dimensional datasets, including structured and unstructured information, to extract insights that traditional methods often miss [17,18]. Studies have demonstrated that ML-based models outperform traditional scoring systems such as SAPS and APACHE in predicting ICU mortality. Models such as gradient boosting machines, convolutional neural networks, and long short-term memory (LSTM) networks have shown significant improvements in prediction accuracy and the potential for real-time application in clinical settings [19-21].

Despite these advancements, challenges remain in translating these models into real-world clinical practice. Many existing models rely on data from the first 24 hours of ICU admission and focus on short- to medium-term outcomes [22-24]. However, ICU mortality often peaks within the first 24 hours and decreases with appropriate management. This highlights the need for dynamic, real-time prediction models that can continuously update risk assessments as the patient’s condition evolves [25,26]. In particular, current ML approaches face limitations in handling the irregular and longitudinal nature of electronic medical record (EMR) data. First, many models rely on manually aggregated features or fixed time-window summarizations, which may overlook fine-grained temporal patterns and evolving physiological trajectories. As a result, critical transitions in patient status may not be adequately captured [27]. Second, the temporal irregularity of EMR data often leads to missing values or asynchronous variable recording. Conventional models usually assume regularly sampled data and require imputation strategies that may introduce bias or degrade predictive accuracy. Robust modeling of both the timing and availability of measurements remains an open challenge in ICU risk prediction [28]. Furthermore, while many models perform well in specific cohorts, their generalizability across diverse clinical settings remains uncertain. Most models lack external validation and require further evaluation in multicenter cohorts.

To address these gaps, we propose developing a dynamic, real-time risk prediction model for ICU patients. This model will leverage the longitudinal, irregular dynamic data commonly found in EMRs, such as vital signs, laboratory results, and continuous medication use [28,29]. Our model is based on a time-aware bidirectional attention-based long short-term memory (TBAL) framework designed to use multisource EMR data to predict the dynamic in-hospital mortality risk of critically ill patients in real time. By incorporating methods such as irregular time interval awareness and attention mechanisms, the model learns dynamic trends and dependencies in longitudinal data to enhance prediction performance. We also aim to cross-validate the model using multicenter public datasets to ensure its generalizability and robustness across different clinical settings. This study seeks to develop a more accurate, dynamic, and interpretable deep learning model for ICU mortality prediction, ultimately supporting clinical decision-making and improving patient outcomes in critical care environments.

## Methods

### Data Sources

**Figure 1:**
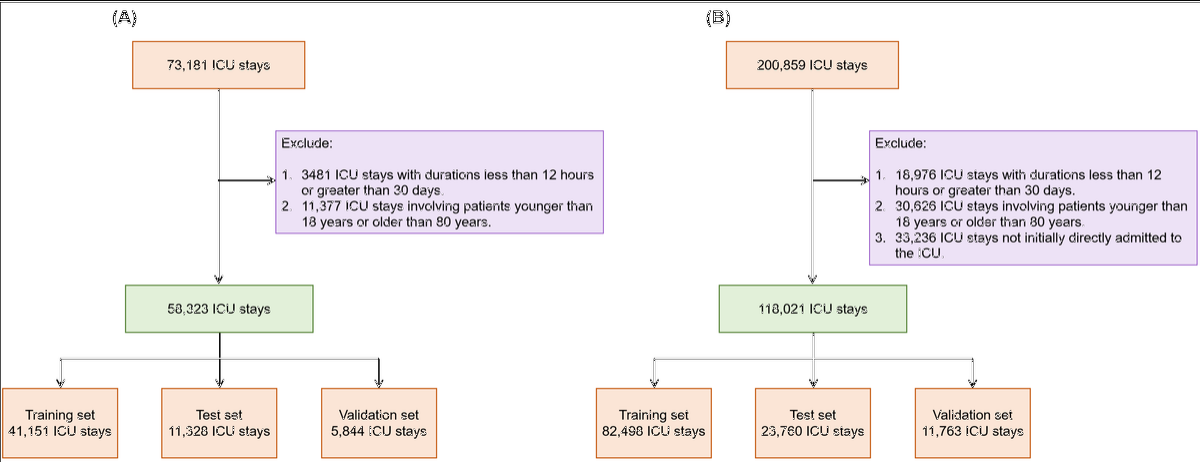
(A) Sample selection process in the MIMIC-IV database and (B) sample selection process in the eICU-CRD database. eICU-CRD: eICU Collaborative Research Database; ICU: intensive care unit; MIMIC-IV: Medical Information Mart for Intensive Care IV.

We used 2 large and well-known public EMR databases: the MIMIC-IV (Medical Information Mart for Intensive Care) database [30] and the eICU Collaborative Research Database (eICU-CRD) [31]. These databases contain critical longitudinal irregular data for patient care, such as physiological measurements, laboratory tests, medications, and fluid outputs. The MIMIC-IV database includes deidentified records of patients treated in the ICU or emergency department at the Beth Israel Deaconess Medical Center in Boston from 2008 to 2019. We extracted data for 73,181 ICU stays, covering 50,920 patients. Each ICU stay in this database is uniquely identified by a “stay_id.” The eICU-CRD database contains records of patients treated in 200 ICU units across the United States between 2014 and 2015. From this database, we extracted 200,859 ICU stays involving 139,367 patients. Each ICU stay is uniquely identified by a “patientunitstayid.” We used ICU stays as the unit of analysis and excluded stays shorter than 12 hours or longer than 30 days. ICU stays of less than 12 hours often lack enough data to evaluate task performance, especially in continuous dynamic prediction tasks. Stays exceeding 30 days usually involve overly complex cases. We also excluded patients younger than 18 or older than 80 years due to their smaller sample sizes, which could reduce the representativeness of the results. Finally, we retained 58,323 ICU records from the MIMIC-IV database and 118,021 ICU records from the eICU-CRD database. Figure 1 provides a detailed overview of the sample selection process for both databases.

### Variable Preprocessing

To standardize clinical concepts across the 2 databases, we used 2 widely recognized resources: eicu-code [32] for eICU-CRD and mimic-code [33] for MIMIC-IV. These resources were used to map and unify variables and codes in the 2 databases, creating consistent clinical definitions and ensuring data comparability [34].

Our data included patient demographics, medical history, laboratory test results, vital signs, medication use, urine output, and mechanical ventilation status. Except for demographics, all other data were longitudinal and irregular. To model these irregular time series, we followed the approach of the recently proposed Electronic Medical Record Longitudinal Irregular Data Preprocessing (EMR-LIP) framework, which is specifically designed for handling longitudinal, irregular EMR data. Following the recommendations of EMR-LIP, we consulted with clinicians and constructed a variable dictionary that defined the data type, aggregation method, and imputation strategy for each variable. Detailed variable dictionaries are provided in Tables S5 and S6 in Multimedia Appendix 1. Of note is that these aggregation and imputation methods were designed based on the characteristics of different clinical variables, including their value types and measurement methods. Therefore, they are highly aligned with clinical practice. Although some studies suggest using 0 as a placeholder to allow the model to learn missing patterns automatically, our approach may offer better interpretability.

To align the dynamic variables over time, we discretized the timeline into 1-hour intervals, starting from ICU admission until discharge. Dynamic variables were resampled to match these time points. For each variable *x_d_* at time point *t_i_*, if multiple observations occurred within the interval [*t_i_*–0.5, *t_i_*+0.5], we aggregated them using the method specified in the variable dictionary, for example, using the median for numerical variables and the mode for categorical variables. If no observations were present, the value was marked as missing. We introduced a mask matrix to track the observation status of each variable at each time point. For a variable *x_d_* at time *t_i_*, *m_t,d_*=1 if an observation was available, and *m_t,d_*=0 otherwise. This mask matrix served as a binary indicator of the observation pattern over time for all dynamic variables. Additionally, to retain information about the intervals between consecutive observations after resampling, we computed a time interval vector *δ_t_* that captures the time since the last observation for each variable. The specific calculation method is described in Multimedia Appendix 1.

During the missing value imputation stage, we applied different methods based on the type and timing of the missing data, as defined in the variable dictionary. For example, for variables missing their first observation, we used the Last Observation Carried Forward method. If no observations were available during the entire ICU stay, we used the median (for numerical variables) or mode (for categorical variables) from the training set. For other missing values, we used linear interpolation for numerical variables, and assigned a separate “missing” category for categorical variables. If no variables had observations at a specific time point *t_i_* that time point was removed.

### Model Development

Our goal was to build a dynamic and continuous risk assessment model for predicting in-hospital mortality in ICU patients. To achieve this, we divided the tasks into static prediction tasks triggered at key time points and dynamic prediction tasks triggered continuously [15,35]. The key difference between the 2 tasks lies in their prediction time windows. For static tasks, the prediction time window is fixed, such as the period between ICU discharge and hospital discharge. In contrast, for dynamic tasks, the prediction time window is continuously updated, such as predicting mortality within the next 24 hours at each time point.

In the static tasks, we set the 12th hour after ICU admission as the key time point. The tasks included predicting 12-hour to 1-day mortality, 12-hour to 2-day mortality, 12-hour to 4-day mortality, 12-hour to 7-day mortality, in-hospital mortality after 12 hours, and ICU length of stay greater than 2 days. For dynamic tasks, we evaluated mortality within the next 24 hours at each hourly interval.

The baseline model used in this study was an LSTM network, a recurrent neural network architecture well-suited for time-series forecasting [36]. LSTM neurons operate through 3 main gates: the input gate, which controls the flow of new data into the model; the forget gate, which determines whether to discard irrelevant information; and the output gate, which regulates the use of updated information for the current prediction. These mechanisms enable LSTM networks to effectively learn and retain patterns in sequential data, making them an ideal choice for processing longitudinal datasets.

To enhance the performance of the baseline LSTM model, we developed a TBAL network. The TBAL model extends the capabilities of LSTM by incorporating 2 key features. First, it uses a bidirectional LSTM structure to capture temporal dependencies in both forward and backward time directions, enabling the model to learn more comprehensive temporal patterns. Second, a time-aware attention mechanism dynamically assigns weights to data at different time points, prioritizing the most relevant information for each prediction.

The TBAL model was specifically designed to handle multivariate time-series data and provide hourly updated predictions, balancing the need for frequent updates with manageable model complexity. By integrating newly accumulated data and learning from evolving temporal trends, the TBAL model outperformed the baseline LSTM in accuracy and interpretability. Additional details about the TBAL model can be found in Multimedia Appendix 1.

We treated each ICU stay as a sample unit, but we grouped the data by patient to avoid data leakage, as some patients had multiple ICU stays. We randomly divided the selected samples from the MIMIC-IV and eICU-CRD databases into training, testing, and validation sets in a 7:2:1 ratio by patient. The training set was used for model training, the validation set for optimal model selection, and the testing set for internal generalizability testing and external cross-validation.

To test the cross-database generalization ability of the model between MIMIC-IV and eICU-CRD, we addressed differences in the variable sets of the 2 databases. We identified 34 common dynamic variables with consistent definitions between the 2 databases. Separate models were trained on the static and dynamic tasks within each database. After training, we evaluated the models on their respective test sets and conducted cross-testing between the 2 databases.

For hyperparameter settings, grid search was avoided. Instead, relatively large values were selected, such as an LSTM hidden size of 512, to ensure sufficient model capacity. L2 regularization and early stopping were applied to prevent overfitting. Detailed hyperparameter settings are provided in Table S9 in Multimedia Appendix 1. The TBAL model has moderate computational requirements and can run on a standard CPU. The total number of parameters in the TBAL model is 727,640, and the memory or GPU usage during inference is approximately 2.79 MB. Overall, TBAL is a lightweight network. On a server with an Intel Xeon Gold 6152 CPU (2.10 GHz, 256 GB RAM; Intel Corporation), the model achieves an inference speed of 35 forward passes per second. Therefore, TBAL is fully capable of supporting real-time prediction with hourly updates in clinical practice.

To address label imbalance, we used different strategies for static and dynamic tasks. In static tasks, where survivors significantly outnumbered nonsurvivors, we implemented balanced sampling during training. This involved setting a fixed number of samples for both minority and majority classes (eg, 200) during each gradient descent step to ensure balanced subsets. Multiple iterations were performed in each epoch to ensure full data use. Over many epochs, the model was exposed to the entire dataset.

For dynamic tasks, balanced sampling was not applicable due to continuous prediction. Instead, we introduced a balance factor in the Cross-Entropy Loss function, assigning different weights to each class to balance their contributions. For binary tasks, the weighted Cross-Entropy Loss formula is:

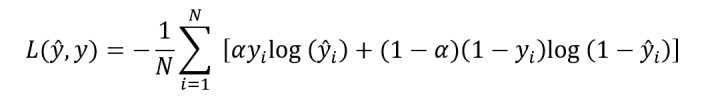



where *α* ∈ [0,1] is the balance factor. This factor adjusts the contributions of positive and negative samples to the loss. In this study, *α* was determined as the inverse of the class proportions, followed by normalization.

### Model Interpretation

We applied the integrated gradients (IG) method to our deep learning model to address the interpretability challenges posed by its black-box nature. IG is a widely recognized technique for feature attribution, quantifying the contribution of each input feature to the model’s output. It achieves this by integrating the gradients of the model’s output concerning the input features along a path from a baseline input to the actual input [37]. This approach ensures that the feature contributions are calculated in a principled manner, based on their incremental effect on the prediction. Unlike other attribution methods, IG satisfies key properties such as completeness and sensitivity, making it a robust choice for understanding model predictions. By leveraging IG, we aim to identify and interpret the features that drive the model’s decision-making process, ensuring a transparent link between input features and predictions. Specifically, for an input *x* and a model *F*, IG is defined as:

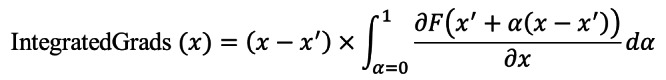



where *x'* is a suitably chosen baseline, and *α* defines the path from the baseline to the input. In practice, the integral is approximated numerically as:

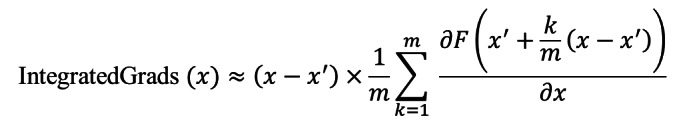



where *m* is the number of steps used for the approximation. The baseline *x'* is chosen as a tensor of zeros. Continuous variables were *z* score normalized, and categorical variables were 1-hot encoded, making the 0 tensor a reasonable baseline for standardized continuous features.

### Model Evaluation

We evaluated the model’s performance using several metrics, including the area under the receiver operating characteristic curve (AUROC), the area under the precision-recall curve (AUPRC), accuracy, recall, precision, and *F*_1_-score. We conducted extensive sensitivity analyses across subgroups defined by gender, age, and race. For the dynamic prediction tasks, we also assessed the model’s performance at different time points.

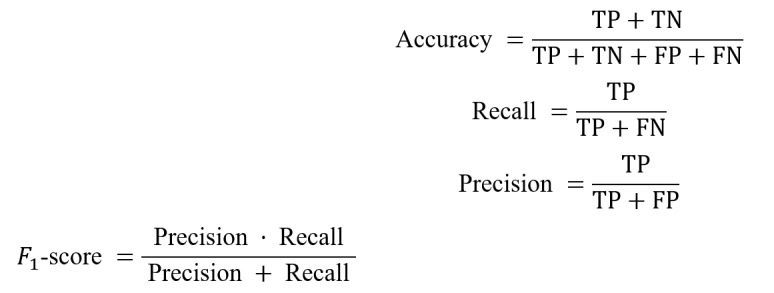



where TP represents true positives, TN represents true negatives, FP represents false positives, and FN represents false negatives, the 95% CIs were estimated using bootstrapping with 1000 samples.

### Ethical Considerations

The MIMIC-IV database was publicly released after receiving approval from the institutional review boards of Beth Israel Deaconess Medical Center and the Massachusetts Institute of Technology in Boston, United States. The eICU-CRD was made publicly accessible after obtaining appropriate institutional review board approvals from 208 hospitals in the United States. Both databases contain fully deidentified data that are publicly available for research purposes. Accordingly, this study was determined to be exempt from further ethical review, and informed consent was waived. No identifiable personal information was accessed or used, and all analyses were performed on anonymized datasets to protect participant privacy and confidentiality. No compensation was provided to any individual, as this study involved secondary analysis of existing, deidentified data.

## Results

To build the model, we included a total of 176,344 ICU stays from the 2 databases, with 58,323 stays from the MIMIC-IV database and 118,021 stays from the eICU-CRD database. Table 1 presents the baseline characteristics of the included patients from both databases, using ICU stays as the unit of analysis. Table S1 in Multimedia Appendix 1 shows the baseline characteristics of the training, testing, and validation sets from both MIMIC-IV and eICU-CRD. Among all included samples, the overall in-hospital mortality rate was 8.1%. The in-hospital mortality rate in the MIMIC-IV database was higher at 9.6% compared to 7.3% in the eICU-CRD database.

**Table 1:** Demographic characteristics of selected samples from different databases.

			Overall		eICU-CRD^a^		MIMIC-IV^b^
ICU^c^ stays, n			176,344		118,021		58,323
Age (years), mean (SD)			58.76 (14.83)		58.71 (14.93)		58.85 (14.61)
**Gender, n (%)**							
	Female	77,044 (43.7)		52,348 (44.4)		24,696 (42.3)	
	Male	99,268 (56.3)		65,641 (55.6)		33,627 (57.7)	
	Other or unknown	32 (0)		32 (0)		0 (0)	
**Race, n (%)**							
	Asian	3696 (2.1)		1953 (1.7)		1743 (3)	
	Black or African American	21,349 (12.1)		14,517 (12.3)		6832 (11.7)	
	Hispanic or Latino	6876 (3.9)		4439 (3.8)		2437 (4.2)	
	White	127,577 (72.3)		88,804 (75.2)		38,773 (66.5)	
	Other or unknown	16,846 (9.6)		8308 (7)		8538 (14.6)	
ICU LoS^d^ (hours), mean (SD)			83.59 (95.93)		84.62 (96.1)		81.52 (95.56)
12h_to_1d mortality, n (%)			1333 (0.8)		718 (0.6)		615 (1.1)
12h_to_2d mortality, n (%)			3224 (1.8)		1929 (1.6)		1295 (2.2)
12h_to_4d mortality, n (%)			5827 (3.3)		3617 (3.1)		2210 (3.8)
12h_to_7d mortality, n (%)			8372 (4.7)		5190 (4.4)		3182 (5.5)
In-hospital mortality, n (%)			14224 (8.1)		8648 (7.3)		5576 (9.6)

^a^eICU-CRD: eICU Collaborative Research Database.

^b^MIMIC-IV: Medical Information Mart for Intensive Care IV.

^c^ICU: intensive care unit.

^d^LoS: length of intensive care unit stay.

In the static prediction tasks triggered at the 12th hour after ICU admission, such as 12-hour to 1-day mortality, 12-hour to 2-day mortality, 12-hour to 4-day mortality, 12-hour to 7-day mortality, in-hospital mortality after 12 hours, and ICU length of stay greater than 2 days, the TBAL model consistently outperformed the baseline LSTM model. For the 12-hour to 1-day mortality task, the AUROC of TBAL reached 95.9 (95% CI 94.2-97.5) in the MIMIC-IV database and 93.3 (95% CI 91.5-95.3) in the eICU-CRD database. For more detailed performance information, see Table 2 and Tables S7 and S8 in Multimedia Appendix 1.

**Table 2:** Performance of the models on various static tasks and continuous dynamic prediction tasks on the internal test set.

Databases and tasks			Triggering^a^		Outcome prevalence (%)		Models		AUROC^b^ (%; 95% CI)		AUPRC^c^ (%; 95% CI)
**MIMIC-IV^d^**											
	12 h to 1 d mortality	12th hour		1.1		TBAL^e^		95.9 (94.2-97.5)		48.5 (43.2-58.3)	
	12 h to 1 d mortality	12th hour		1.1		LSTM^f^		91.2 (87.7-94.3)		33.9 (24.5-42)	
	12 h to 2 d mortality	12th hour		2.2		TBAL		92.9 (91.3-94.7)		45.2 (39.6-49.6)	
	12 h to 2 d mortality	12th hour		2.2		LSTM		91.8 (90.1-93.5)		40.8 (35.8-46.6)	
	12 h to 4 d mortality	12th hour		3.8		TBAL		91.9 (90.5-92.8)		47.4 (42.9-52.1)	
	12 h to 4 d mortality	12th hour		3.8		LSTM		91.5 (90-92.5)		42.5 (37.8-48.3)	
	12 h to 7 d mortality	12th hour		5.5		TBAL		90.1 (89.6-91.3)		44 (41.5-47.7)	
	12 h to 7 d mortality	12th hour		5.5		LSTM		89.7 (88.4-90.9)		40.6 (36.7-43.4)	
	In-hospital mortality	12th hour		9.6		TBAL		88.8 (88.1-89.6)		52.4 (50.4-54.8)	
	In-hospital mortality	12th hour		9.6		LSTM		88.4 (86.9-89.5)		50.4 (47.3-53.6)	
	ICU^g^ LoS^h^ > 2 d	12th hour		82.2		TBAL		80.5 (79.8-81.2)		95.1 (94.8-95.5)	
	ICU LoS > 2 d	12th hour		82.2		LSTM		78.1 (77.2-79.1)		94.5 (94.2-94.9)	
	Death within the next 24 hours	4 hourly		2.2		TBAL		93.6 (93.2-93.9)		41.3 (39.8-42.3)	
	Death within the next 24 hours	4 hourly		2.2		LSTM		93.7 (93.4-94)		42.9 (41.4-44.9)	
**eICU-CRD^i^**											
	12 h to 1 d mortality	12th hour		0.6		TBAL		93.3 (91.5-95.3)		21.6 (16.4-27.9)	
	12 h to 1 d mortality	12th hour		0.6		LSTM		92.8 (90.8-94.7)		16.1 (12.3-21.1)	
	12 h to 2 d mortality	12th hour		1.6		TBAL		91 (89.4-93.4)		30.3 (25.1-35.1)	
	12 h to 2 d mortality	12th hour		1.6		LSTM		90.9 (89.9-92)		27.3 (22.4-32.1)	
	12 h to 4 d mortality	12th hour		3.1		TBAL		89.8 (88.5-90.8)		35.8 (32.6-39.7)	
	12 h to 4 d mortality	12th hour		3.1		LSTM		89.1 (87.6-90)		33.4 (30.9-37.3)	
	12 h to 7 d mortality	12th hour		4.4		TBAL		89 (87.9-90)		39.8 (36.8-43)	
	12 h to 7 d mortality	12th hour		4.4		LSTM		88.4 (87.6-89.4)		36.9 (33.5-40)	
	In-hospital mortality	12th hour		7.3		TBAL		87.1 (86.5-87.8)		44.4 (41.7-46.6)	
	In-hospital mortality	12th hour		7.3		LSTM		86.7 (85.9-87.7)		42.7 (40.6-45.7)	
	ICU LoS > 2 d	12th hour		79.9		TBAL		74.5 (74-75.2)		92.2 (91.9-92.5)	
	ICU LoS > 2 d	12th hour		79.9		LSTM		74.3 (73.8-74.9)		92.1 (91.8-92.4)	
	Death within the next 24 hours	4 hourly		1.8		TBAL		91.9 (91.6-92.1)		50 (49.2-50.7)	
	Death within the next 24 hours	4 hourly		1.8		LSTM		91.5 (91.3-91.7)		44.8 (44.3-45.5)	

^a^The trigger times for these tasks are all relative to the intensive care unit admission time.

^b^AUROC: area under the receiver operating characteristic curve.

^c^AUPRC: area under the precision-recall curve.

^d^MIMIC-IV: Medical Information Mart for Intensive Care IV.

^e^TBAL: time-aware bidirectional attention-based long short-term memory.

^f^LSTM: long short-term memory.

^g^ICU: intensive care unit.

^h^LoS: length of intensive care unit stay.

^i^eICU-CRD: eICU Collaborative Research Database.

**Figure 2:**
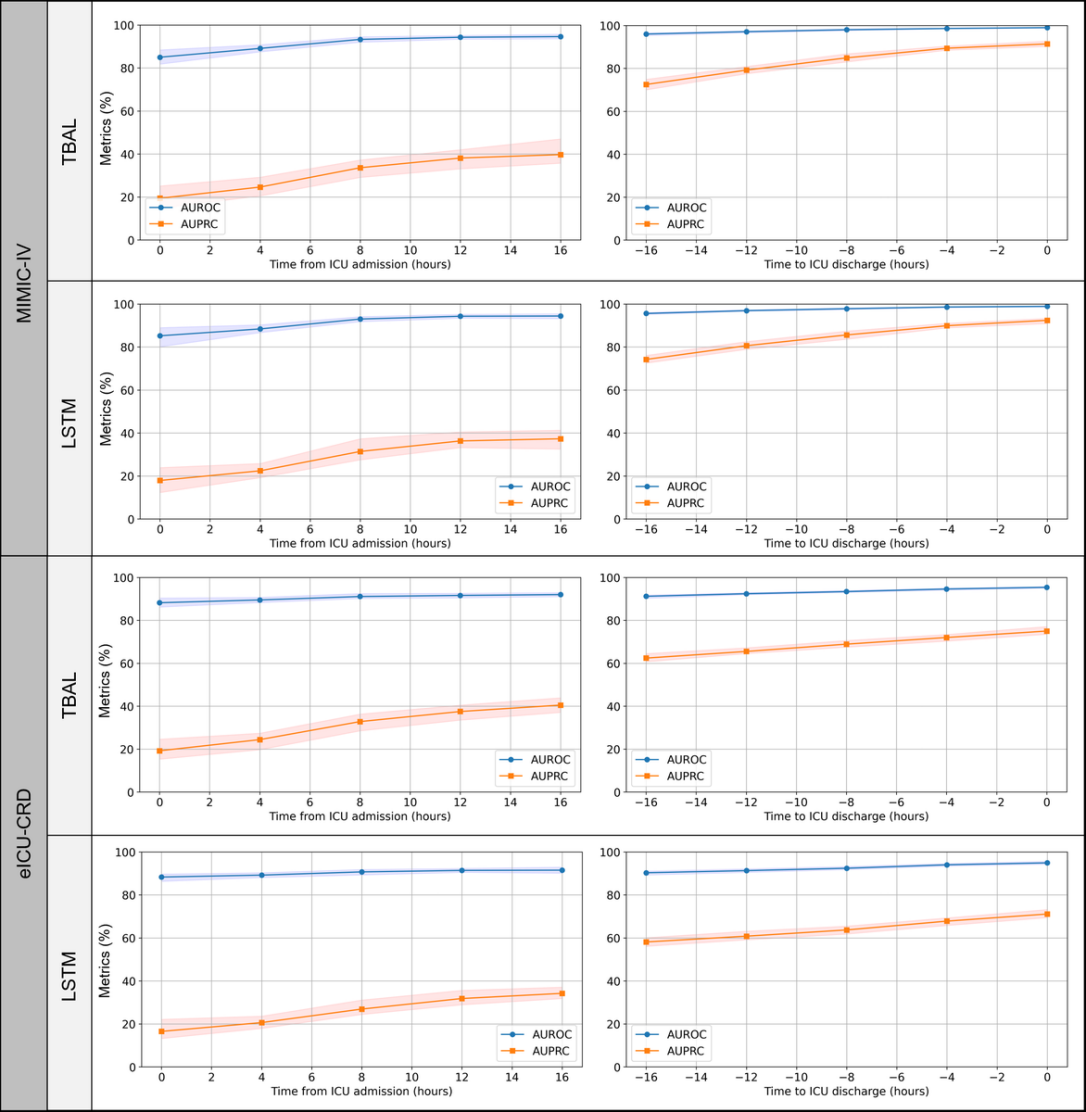
Performance of different models in predicting mortality within the next 24 hours on the internal test set, evaluated every 4 hours throughout the ICU stay. AUPRC: area under the precision-recall curve; AUROC: area under the receiver operating characteristic curve; eICU-CRD: eICU Collaborative Research Database; ICU: intensive care unit; MIMIC-IV: Medical Information Mart for Intensive Care IV; LSTM: long short-term memory; TBAL: time-aware bidirectional attention-based long short-term memory.

In the dynamic continuous mortality risk assessment tasks, the performance of TBAL was comparable to that of the LSTM model. Across the entire ICU stay, the AUROC reached 93.6 (95% CI 93.2-93.9) in the MIMIC-IV database and 91.9 (95% CI 91.6-92.1) in the eICU-CRD database. Further analysis of the model’s performance at 4-hour intervals after ICU admission revealed that the performance was not uniform throughout the ICU stay. Both the TBAL and LSTM models showed an initially lower performance, which gradually improved over time. By the time of ICU discharge, the AUROC and AUPRC of the TBAL model reached 98.9 (95% CI 98.6-99.2) and 92.1 (95% CI 90.6-93.7) in the MIMIC-IV database and 95.4 (95% CI 95-95.9) and 71.3 (95% CI 69.3-73.8) in the eICU-CRD database. Overall, the performance of the model in the MIMIC-IV database was consistent with its performance in the eICU-CRD database, although differences in AUPRC were observed in some tasks. These differences may be related to variations in the variable sets used in the 2 databases. Figure 2 and Tables S2 and S3 in Multimedia Appendix 1 provide a detailed performance comparison for the dynamic task of predicting mortality within the next 24 hours at various time points.

**Figure 3:**
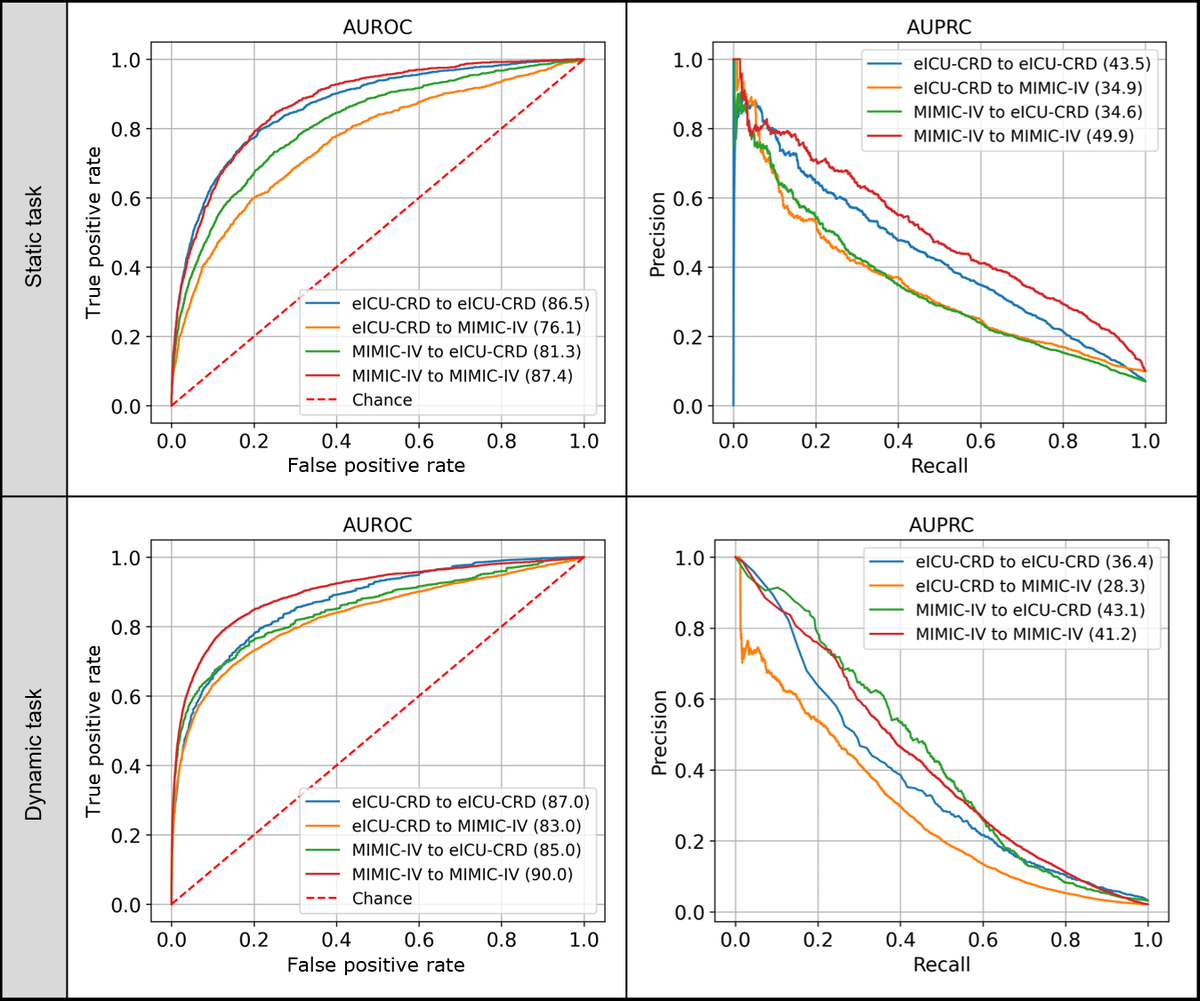
Cross-generalization performance test results of the TBAL model on data from the MIMIC-IV and eICU-CRD databases. Static task refers to predicting in-hospital mortality triggered 12 hours after ICU admission. Dynamic task refers to predicting mortality within the next 24 hours, triggered every 4 hours after ICU admission. AUPRC: area under the precision-recall curve; AUROC: area under the receiver operating characteristic curve; eICU-CRD: eICU Collaborative Research Database; ICU: intensive care unit; MIMIC-IV: Medical Information Mart for Intensive Care IV; TBAL: time-aware bidirectional attention-based long short-term memory.

In cross-validation experiments, the AUROC for transferring the model from MIMIC-IV to eICU-CRD was 0.813, while transferring from eICU-CRD to MIMIC-IV resulted in an AUROC of 0.761. For dynamic tasks, the performance of MIMIC-IV and eICU-CRD was also very close, with AUROC values of 0.9 and 0.87, respectively. In cross-generalization testing for dynamic tasks, transferring from MIMIC-IV to eICU-CRD achieved an AUROC of 0.85, while transferring from eICU-CRD to MIMIC-IV achieved an AUROC of 0.83. Figure 3 and Table S4 in Multimedia Appendix 1 summarize the cross-generalization performance results of the TBAL model using data from both the MIMIC-IV and eICU-CRD databases.

In the subgroup analysis, we evaluated the performance of the TBAL model for both static and dynamic tasks across different genders, age groups, and races. Table 3 shows the detailed results of the subgroup analysis. For age groups, the model performed better in predicting outcomes for patients aged younger than 65 years compared to those aged 65 years and older, regardless of whether the task was static or dynamic. For gender, the model showed balanced performance between males and females in both types of tasks. For race, we observed slight differences in performance between racial groups. However, the 95% CIs for AUROC and AUPRC overlapped across all racial groups, suggesting that these differences were not statistically significant. Overall, the model demonstrated consistent performance across different racial groups.

**Table 3:** Summary of the performance analysis of the TBAL^a^ model across different subgroups.

Database and subgroup				Static task^b^ (95% CI)					Dynamic task^c^ (95% CI)		
				AUROC^d^		AUPRC^e^		AUROC			AUPRC
**MIMIC-IV^f^**											
	**Age**										
		<65	88.7 (87.5-90.5)		47.8 (42.3-51.5)		91.4 (90.8-91.9)			41.6 (39.4-43.7)	
		≥65	85.6 (84.3-86.7)		53 (49.2-56.2)		88.4 (87.8-89.2)			41.2 (39.1-42.8)	
	**Gender**										
		Female	87.4 (85.9-89.1)		48.8 (44.3-52.2)		90.6 (89.8-91.4)			41.8 (39.8-43.7)	
		Male	87.3 (85.9-88.5)		50.8 (48-53.7)		89.6 (88.9-90.2)			41 (38.8-42.7)	
	**Race**										
		Asian	82.5 (76.4-89.2)		50.2 (36.4-63.7)		92.5 (90.6-94.4)			48.9 (41.4-57.1)	
		Black or African American	88.6 (86.1-90.8)		42.7 (34.4-51.2)		90.8 (88.8-92.5)			38 (33.5-43.1)	
		Hispanic or Latino	86.2 (81.4-91.1)		45 (31.1-59.4)		92.7 (90.9-94.2)			45.4 (36.6-53.4)	
		White	87.1 (85.9-88.4)		47.7 (43.3-51.8)		89.6 (89-90.3)			39.5 (37.4-41.3)	
		Other or unknown	88.9 (86.8-91.6)		62.8 (56.8-70)		89.8 (88.5-90.8)			46.2 (43.7-49)	
**eICU-CRD^g^**											
	**Age**										
		<65	88.1 (87.3-89.3)		42.3 (39.9-45.8)		90.4 (90-90.7)			38.3 (37.1-39.1)	
		≥65	84.6 (83.4-86)		45.8 (42.8-49.2)		83.7 (83.3-84.2)			35.5 (34.5-36.7)	
	**Gender**										
		Female	86.5 (84.9-87.8)		43.5 (39.8-47.9)		86 (85.5-86.3)			36.6 (35.6-37.7)	
		Male	86.6 (85.4-87.7)		43.7 (40.9-47.3)		87.7 (87.4-88.1)			36.2 (35.4-37.2)	
	**Race**										
		Asian	83.2 (72.1-93.1)		36 (20-55.7)		80.7 (78.9-82.4)			36.3 (32.2-40.2)	
		Black or African American	86 (83.6-88.5)		39.9 (33.4-47.7)		87.7 (87-88.6)			41 (39.3-42.9)	
		Hispanic or Latino	89.5 (86-92.6)		51.2 (40.2-63.2)		85.4 (83.9-86.9)			31.7 (27.9-34.5)	
		White	86.4 (85.5-87.5)		44.9 (42.3-47.3)		87.3 (87-87.6)			36.3 (35.5-37.1)	
		Other or unknown	88 (86.1-90.5)		44.2 (36.9-53.2)		86.2 (85.2-87.2)			37.4 (34.4-40.9)	

^a^TBAL: time-aware bidirectional attention-based long short-term memory.

^b^Static task: predicting in-hospital mortality triggered at the 12th hour after intensive care unit admission.

^c^Dynamic task: predicting death within the next 24 hours triggered every 4 hours.

^d^AUROC: area under the receiver operating characteristic curve.

^e^AUPRC: area under the precision-recall curve.

^f^MIMIC-IV: Medical Information Mart for Intensive Care IV.

^g^eICU-CRD: eICU Collaborative Research Database.

**Figure 4:**
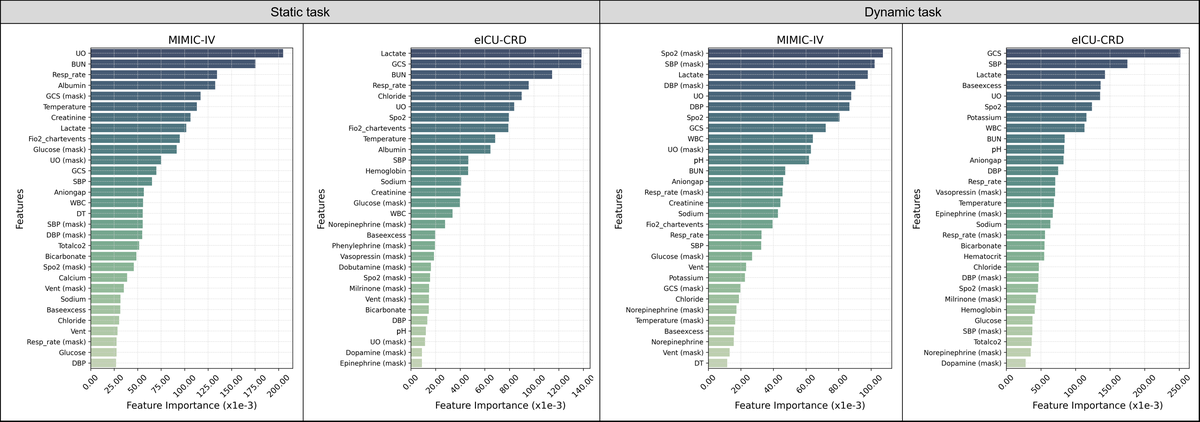
Feature importance ranking for static and dynamic tasks based on the TBAL model. Static task refers to predicting in-hospital mortality triggered 12 hours after ICU admission. Dynamic task refers to predicting mortality within the next 24 hours, triggered every 4 hours after ICU admission. BUN: blood urea nitrogen; DBP: diastolic blood pressure; DT: delta time; eICU-CRD: eICU Collaborative Research Database; Fio2: fraction of inspired oxygen; GCS: Glasgow Coma Scale; ICU: intensive care unit; MIMIC-IV: Medical Information Mart for Intensive Care IV; Resp: respiratory; SBP: systolic blood pressure; SpO2: oxygen saturation; TBAL: time-aware bidirectional attention-based long short-term memory; UO: urine output; WBC: white blood cell count.

We used the IG algorithm to calculate the importance of variables for each patient in both static and dynamic prediction tasks based on the TBAL model. Figure 4 displays the ranked importance of these variables. In static tasks, the variable importance rankings in the MIMIC-IV and eICU-CRD databases showed consistent patterns. In the MIMIC-IV database, blood urea nitrogen, urine output, respiratory rate, lactate, and body temperature were ranked as highly important. Similarly, in the eICU-CRD database, lactate, Glasgow Coma Scale, blood urea nitrogen, respiratory rate, and urine output were also ranked highly. Overall, the top 20 variables in both databases included many shared physiological and laboratory measures. In dynamic tasks, the variable importance rankings also showed a high degree of consistency between the 2 databases. In the MIMIC-IV database, SpO_2_ (oxygen saturation), systolic blood pressure (SBP), lactate, and diastolic blood pressure were among the most important variables. In the eICU-CRD database, Glasgow Coma Scale, SBP, lactate, base excess, and urine output were ranked highly. Compared to static tasks, dynamic tasks highlighted the importance of variables related to vasopressor use (eg, norepinephrine or vasopressin), which were consistently ranked in the top 20 in both databases, indicating their relevance for predicting dynamic mortality risk. Tables S10-S17 in Multimedia Appendix 1 present the IG values of the top 20 features across different tasks in various subgroups.

**Figure 5:**
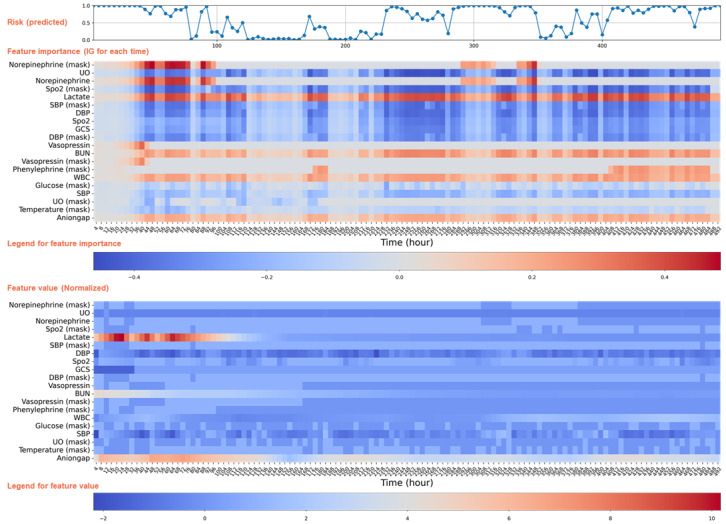
Case analysis of personalized continuous dynamic mortality risk assessment. The top panel shows the TBAL model’s dynamic mortality risk predictions for the patient at 4-hour intervals after ICU admission. The middle panel displays the IG values of the top 20 features most associated with mortality for this patient at each prediction time point during the ICU stay. The bottom panel shows how the values of these top 20 features changed over time. BUN: blood urea nitrogen; DBP: diastolic blood pressure; GCS: Glasgow Coma Scale; ICU: intensive care unit; IG: integrated gradients; SBP: systolic blood pressure; SpO2: peripheral capillary oxygen saturation; TBAL: time-aware bidirectional attention-based long short-term memory; UO: urine output; WBC: white blood cell count.

Figure 5 shows the real-time dynamic mortality risk predictions for a patient who died during hospitalization, providing a comprehensive view of how the patient’s risk changed over time. The top panel displays the predicted risk trajectory, showing fluctuations in mortality risk at hourly intervals. These dynamic changes reflect the model’s sensitivity to evolving clinical conditions, such as critical interventions or physiological deterioration. The middle panel shows the IG values of the top 20 features ranked by their global average absolute IG scores. It reveals how the contribution of each variable to the predicted risk changes over time. Notably, norepinephrine use, including both its presence (mask) and dosage, shows consistently high attribution scores during periods of elevated risk. This suggests a strong association between vasopressor use and mortality risk as an indicator of hemodynamic instability. Other features, such as elevated lactate levels, low SBP, and persistently low urine output, also have strong and time-specific effects during clinical deterioration. The bottom panel displays the normalized values of these top features over time, allowing a direct comparison between model attributions and actual clinical trends. For example, the sharp increase in lactate level occurred almost at the same time as the rise in predicted risk. The IG scores for anion gap also gradually increased as its values rose. This shows how the model integrates both feature presence and temporal patterns to generate risk predictions. In general, it is helpful to first identify time points and variables with strong color intensity in the feature importance map and then examine their corresponding values in the feature value panel to assess abnormal patterns. However, due to the complex temporal dependencies and interactions among variables captured by the model, the importance of a feature may not always be intuitively interpretable.

## Discussion

### Principal Findings

In this study, we developed a dynamic mortality risk assessment model focused on the ICU setting. The model provides risk assessments at key time points after ICU admission and continuous dynamic mortality risk evaluations throughout the ICU stay, enabling personalized risk alerts. It was trained and tested on dynamic variables from the MIMIC-IV and eICU-CRD databases and demonstrated strong generalizability. The model updates predictions hourly, providing personalized forecasts that improve over time. By the time of ICU discharge, the model achieved an AUROC of 98.9 in the MIMIC-IV database and 95.4 in the eICU-CRD database. Our model is interpretable, offering population-level and individual-level rankings of the most important dynamic features associated with mortality risk. At the individual level, we observed that the IG values of features change over time, reflecting the evolving mortality risk as the treatment progresses. This adaptability makes our model more useful than traditional scoring systems, such as SAPS or APACHE, which generate a single score based on data from the first day of ICU admission [24,38-42]. These findings support the importance of continuously updating decision support tools to adapt to changing clinical conditions and provide real-time guidance to clinicians [39]. This dynamic tool could be more effective than the static scores currently used in ICU settings [38]. Early clinical decisions, such as whether to initiate treatment and how aggressively to treat a patient, differ significantly from later decisions, such as whether to withdraw life-sustaining therapy [43]. For example, we found that after aggressive treatment, a patient’s mortality risk might decrease during a certain period. This finding aligns with clinical expectations. Overall, we observed complex interactions among features over time, emphasizing the need for decision support tools based on real-time ML. These features interact in complex, nonlinear ways, unlike the pairwise or 3-way interactions commonly modeled in generalized linear models. Although the data are longitudinal and irregular, the TBAL architecture enables learning from complex sequence patterns while modeling multidimensional interactions between variables. However, this complexity also makes clinical interpretation of the results more challenging, requiring cautious use of the model in practice. In addition to technical considerations, the ethical implications of real-time risk prediction should not be overlooked. Issues such as patient privacy, informed consent, and the responsible use of predictive models in clinical workflows must be carefully addressed to ensure safe and equitable deployment.

### Comparison With Prior Work

From a performance perspective, our model achieved an AUROC of up to 95.9 for predicting in-hospital mortality at the 12th hour after ICU admission, significantly outperforming traditional severity scoring systems. For example, in a multiethnic US cohort, APACHE-IV achieved an AUROC of 86 [27], while SAPS-III showed an AUROC of 79 in an external surgical ICU validation study [44]. This improvement is likely due to the additional benefits of using RNN-based deep learning models, which are effective at capturing longitudinal patterns [38], and the incorporation of multiple data sources, providing richer information through a larger feature set. In previous studies on in-hospital mortality risk assessment, Moreno et al [45] reported an AUROC of 0.814 for the SAPS-III model in a cohort from Northern Europe. However, external validation in Denmark showed a performance drop to an AUROC of 0.69 (95% CI 0.63-0.75) [46]. This decline is similar to the performance drop observed in our study when transferring a model trained on the MIMIC-IV database to the eICU-CRD database. This decline may be due to distribution bias in routinely collected data, which can vary across different centers, affecting the model’s generalization performance. The aggregation and imputation methods were selected based on the EMR-LIP framework and aligned with clinical practice, which may enhance the model’s robustness and generalizability by better reflecting real-world data patterns. Integrating domain expertise into the preprocessing design helps tailor the pipeline to clinical realities, which in turn supports model stability and generalization across different settings. Ensuring consistency in preprocessing steps further safeguards model performance during deployment and external validation. Standardized preprocessing across datasets may also help reduce the impact of distribution bias on external performance. Although missing data is unavoidable in longitudinal irregular datasets, our recommended imputation methods mimic medical reasoning. For example, a missing pH value might indicate that a clinician decided further analysis was unnecessary. In such cases, carrying forward the most recent value for imputation often has clinical relevance. The TBAL model effectively learns information from irregular time intervals and captures the relative importance of features at different time points. These abilities are key to its performance improvement [47,48]. The results from external validation experiments show that using data from similarly homogeneous settings, such as MIMIC-IV or eICU-CRD, allows the model to be practical for use with patients typically encountered by clinicians in their daily work.

### Subgroup Analysis and Algorithmic Bias

Gender and racial biases have played a significant role in the recent critical discussions about biased decisions made by ML models [27]. Such biases can also be observed in the predictions made by the proposed TBAL model. In our subgroup analysis, we found a performance bias related to age. The AUROC for patients aged younger than 65 years was significantly higher than for those aged 65 years and older. This may be because older patients tend to have more complex conditions, which makes predictions more challenging for the model. For race, we observed that the AUROC for Asian patients was relatively low in both the MIMIC-IV and eICU-CRD databases. However, in the dynamic continuous prediction tasks within MIMIC-IV, the AUROC for Asian patients was relatively higher. We believe these differences are due to factors such as sample size, the balance of labels within subgroups, and sample representativeness [49]. These factors differ fundamentally from the performance bias observed in the age subgroups. For gender subgroups, the model showed excellent consistency across different types of tasks and databases. A potential solution to address subgroup performance bias is to locally retrain the model using a more diverse dataset if the model was pretrained on biased data. Until then, it is essential to continuously evaluate predictions, especially considering that cohort compositions may change in the future.

### Model Interpretability and Ethical Considerations

Concerning model interpretability, the 2018 European General Data Protection Regulation raised concerns about black-box predictions. It states that individuals have the right to receive “meaningful information about the logic involved, as well as the significance and envisaged consequences” when automated decisions are used [50,51]. One advantage of our model is that the IG method allows us to explain the importance of features associated with ICU mortality both at the population level and for individual patients at any given time. This enables the model to support clinical decision-making by providing real-time information about a patient’s mortality risk and the key features associated with their survival. Our findings highlight the importance of continuously updating mortality predictions. Patient mortality risk can change dynamically, and the contributing features can also shift over time. However, the IG method only identifies correlations between features and prediction outcomes without inferring causality. For example, the model identifies vasopressor use as positively correlated with increased mortality risk. This correlation likely reflects the fact that patients receiving vasopressors are in critical condition, even though the medication itself is intended to improve their state. While the importance of vasopressor use is correctly identified, the findings cannot directly inform treatment decisions. Many ML methods remain opaque. Although we have made progress by using the IG method to identify and measure the factors driving predictions, IG values cannot address algorithmic bias. Algorithmic bias is a critical issue in ML prediction models. It arises because these models lack an underlying causal structure and rely entirely on historical human behaviors to make predictions. The absence of causal structure means the model may perform poorly for minority groups, as it has limited exposure to such patients during training.

### Limitations and Future Directions

This study has several limitations. First, although the model was trained and validated using 2 large publicly available ICU datasets (MIMIC-IV and eICU-CRD), they may not fully represent ICU populations in other geographic regions or health care systems. MIMIC-IV is derived from a single academic medical center, while eICU-CRD includes data from multiple hospitals with different clinical practices. Differences in care delivery, documentation, and data collection could lead to inconsistencies and affect model generalizability. Despite harmonization efforts, residual heterogeneity may remain. Therefore, further validation in prospective and non-US ICU settings is necessary to confirm the model’s applicability in broader clinical contexts. Additionally, the use of an all-0 baseline in integrated gradients may not be optimal for 1-hot encoded categorical features, as it does not correspond to a valid clinical category and could bias attribution results.

### Conclusion

In summary, we developed an interpretable TBAL model for the dynamic real-time assessment of mortality risk in ICU patients. The model was trained, internally validated, and cross-validated externally using the MIMIC-IV and eICU-CRD databases. It demonstrated significantly better performance compared to traditional scoring systems and the baseline LSTM model. As the ICU stay progresses, the predictive performance of the model improves over time. Additionally, the model captured dynamic changes in both mortality risk and feature importance over time, offering insights that are not available from existing static prognostic scoring systems. However, before being used as a bedside tool, the model’s results need to be validated in randomized clinical trials.

## Appendix

Multimedia Appendix 1 Additional data and tables.

## Abbreviations

APACHE: Acute Physiology and Chronic Health Evaluation
AUPRC: area under the precision-recall curve
AUROC: area under the receiver operating characteristic curve
eICU-CRD: eICU Collaborative Research Database
EMR: electronic medical record
EMR-LIP: Electronic Medical Record Longitudinal Irregular Data Preprocessing
ICU: intensive care unit
IG: integrated gradient
LSTM: long short-term memory
MIMIC-IV: Medical Information Mart for Intensive Care IV
ML: machine learning
SAPS: Simplified Acute Physiology Score
SBP: systolic blood pressure
SpO2: oxygen saturation
TBAL: time-aware bidirectional attention-based long short-term memory

## Data Availability

The data used in this study can be accessed on the web [52] by registering and submitting a request. The complete preprocessing code for the MIMIC-IV and eICU-CRD databases used in this study is publicly available [53].
