# Supplementary material for: Development and Validation of a Dynamic Real-Time Risk Prediction Model for Intensive Care Units Patients Based on Longitudinal Irregular Data: Multicenter Retrospective Study
Source: J Med Internet Res. 2025 Apr 23;27:e69293. doi: 10.2196/69293 (PMC12059492; doi:10.2196/69293)
Supplement: Multimedia Appendix 1 [file jmir_v27i1e69293_app1.docx]

**Supplementary**

### Detailed Introduction of the TBAL Model

We selected Long Short-Term Memory (LSTM) and Attention-based Bidirectional Long Short-Term Memory (Bi-LSTM) models to test the performance in various tasks. LSTM networks are designed to address the vanishing gradient problem in traditional RNNs, allowing them to learn dependencies over long time intervals.

Let $X\in\mathbb{R}^{T\times D}$ represent the multivariate time series of a single sample, where $T$ is the length of the series and $D$ is the number of variables. The notation $x_{t,:}=$ $\left[ x_{t,1},x_{t,2},\ldots,x_{t,D} \right]$ denotes the feature vector at time $t$, and $x_{:,d}=\left[ x_{1,d},x_{2,d},\ldots,x_{T,d} \right]$ represents the univariate time series for the $d$-th variable. Additionally, $s_{t}$ denotes the timestamp at time $t$, where $t$ serves as an index.

Multivariate time series $X$ often exhibit irregularities, such as missing data. To address this, we introduce $m_{t}=\left[ m_{t,1},m_{t,2},\ldots,m_{t,D} \right]$, the missingness pattern at time $t$, where $m_{t,d}=1$ indicates that data for the $d$-th variable at $t$ is present, and $m_{t,d}=0$ indicates a missing value. The input to the LSTM cell is augmented to include both $x_{t}$ and $m_{t}$, enabling the model to account for missingness patterns during sequence modeling.

The LSTM unit processes the augmented input $\left[ x_{t};m_{t} \right]$ to compute the hidden state $h_{t}$ and cell state $c_{t}$. The computations are as follows:

$$\begin{matrix} f_{t} & =\sigma\left( W_{f}\cdot\left[ h_{t-1},x_{t},m_{t} \right]+b_{f} \right) \\ i_{t} & =\sigma\left( W_{i}\cdot\left[ h_{t-1},x_{t},m_{t} \right]+b_{i} \right) \\ C_{t} & =tanh\left( W_{C}\cdot\left[ h_{t-1},x_{t},m_{t} \right]+b_{C} \right) \\ c_{t} & =f_{t}*c_{t-1}+i_{t}*C_{t} \\ o_{t} & =\sigma\left( W_{o}\cdot\left[ h_{t-1},x_{t},m_{t} \right]+b_{o} \right) \\ h_{t} & =o_{t}*tanh\left( c_{t} \right) \end{matrix}$$

To handle irregular time intervals, we introduce a time-aware encoding module that computes a time-aware vector $\gamma_{t}$ based on the variable-wise time interval $\delta_{t}=[\delta_{t}^{1},\delta_{t}^{2},\ldots,\delta_{t}^{D}]$, where each element is defined as:

$$\delta_{t}^{d}=\left\{ \begin{matrix} s_{t}-s_{t-1}+\delta_{t-1}^{d}, & t>1,\text{ }m_{t-1}^{d}=0 \\ s_{t}-s_{t-1}, & t>1,\text{ }m_{t-1}^{d}=1 \\ 0, & t=1 \end{matrix} \right.$$

This formulation accumulates time gaps for variables with consecutive missing values, allowing the model to track the actual elapsed time since the last observed measurement for each variable.

The time-aware vector $\gamma_{t}$ is then computed as:

$$\gamma_{t}=exp\left\{ -max\left( 0,\text{ }W_{\gamma}\odot\delta_{t}+b_{\gamma} \right) \right\}$$

where $\odot$ denotes element-wise multiplication, and $W_{\gamma}$, $b_{\gamma}$ are learnable parameter vectors. This vectorized formulation ensures that the model can differentially discount outdated features based on how long they have been missing.

The hidden state $h_{t}$ is then augmented by concatenating it with the time-aware vector $\gamma_{t}$:

$$h_{t}^{\text{updated}}=[h_{t};\gamma_{t}]$$

This enriched hidden state $h_{t}^{\text{updated }}$ incorporates both temporal and feature-level information.

The TBAL model further enhances this representation by integrating an attention mechanism, which assigns weights to each timestep's updated hidden state based on its relevance to the prediction task:

$$\begin{matrix} u_{t} & =tanh\left( W_{u}\cdot h_{t}^{\text{updated }}+b_{u} \right) \\ \alpha_{t} & =\frac{exp\left( u_{t} \right)}{\sum_{t^{'}} exp\left( u_{t^{'}} \right)} \\ h_{\text{weighted }} & =\sum_{t} \alpha_{t}\cdot h_{t}^{\text{updated }} \end{matrix}$$

Here, $W_{u}$ and $b_{u}$ are the weights and biases of the attention layer, $\alpha_{t}$ are the attention weights, and $h_{\text{weighted }}$ is the final context vector summarizing the sequence.

By incorporating $m_{t}$ directly into the LSTM computations and leveraging a time-aware encoding module for $\gamma_{t}$, the TBAL model effectively handles missing data and irregular time intervals. This architecture improves the ability to model multivariate time series and generate robust, interpretable predictions, making it well-suited for complex clinical datasets.

**Supplemental Tables**

**Table S1**. Baseline characteristics of different datasets in the MIMIC-IV and eICU-CRD databases

|  |  | Train | Test | Valid |
| --- | --- | --- | --- | --- |
| **MIMIC-IV** | | | | |
| n |  | 41151 | 11328 | 5844 |
| Age, years (mean (SD)) |  | 58.92 (14.57) | 58.73 (14.74) | 58.63 (14.70) |
| Gender (%) | Female | 17550 (42.6) | 4760 (42.0) | 2386 (40.8) |
|  | Male | 23601 (57.4) | 6568 (58.0) | 3458 (59.2) |
|  | Other/Unknown | 0 ( 0.0) | 0 ( 0.0) | 0 ( 0.0) |
| race (%) | Asian | 1200 ( 2.9) | 365 ( 3.2) | 178 ( 3.0) |
|  | Black/African American | 4716 (11.5) | 1401 (12.4) | 715 (12.2) |
|  | Hispanic/Latino | 1646 ( 4.0) | 532 ( 4.7) | 259 ( 4.4) |
|  | Other/Unknown | 5992 (14.6) | 1704 (15.0) | 842 (14.4) |
|  | White | 27597 (67.1) | 7326 (64.7) | 3850 (65.9) |
| ICU LoS, hours (mean (SD)) |  | 81.47 (96.07) | 81.54 (93.42) | 81.84 (96.09) |
| 12h_to_1d mortality (%) |  | 434 ( 1.1) | 128 ( 1.1) | 53 ( 0.9) |
| 12h_to_2d mortality (%) |  | 914 ( 2.2) | 248 ( 2.2) | 133 ( 2.3) |
| 12h_to_4d mortality (%) |  | 1558 ( 3.8) | 430 ( 3.8) | 222 ( 3.8) |
| 12h_to_7d mortality (%) |  | 2246 ( 5.5) | 624 ( 5.5) | 312 ( 5.3) |
| In-hospital mortality (%) |  | 3893 ( 9.5) | 1122 ( 9.9) | 561 ( 9.6) |
| **eICU-CRD** | | | | |
| n |  | 123649 | 35088 | 17607 |
| Age, years (mean (SD)) |  | 58.82 (14.81) | 58.72 (14.80) | 58.41 (15.01) |
| Gender (%) | Female | 54185 (43.8) | 15101 (43.0) | 7758 (44.1) |
|  | Male | 69437 (56.2) | 19984 (57.0) | 9847 (55.9) |
|  | Other/Unknown | 32 ( 0.0) | 27 ( 0.0) | 3 ( 0.0) |
| Race (%) | Asian | 2582 ( 2.1) | 738 ( 2.1) | 376 ( 2.1) |
|  | Black/African American | 14756 (11.9) | 4455 (12.7) | 2138 (12.1) |
|  | Hispanic/Latino | 4777 ( 3.9) | 1406 ( 4.0) | 693 ( 3.9) |
|  | Other/Unknown | 11785 ( 9.5) | 3415 ( 9.7) | 1646 ( 9.3) |
|  | White | 89749 (72.6) | 25074 (71.5) | 12754 (72.4) |
| ICU LoS, hours (mean (SD)) |  | 83.39 (95.61) | 84.31 (97.37) | 83.56 (95.30) |
| 12h_to_1d mortality (%) |  | 937 ( 0.8) | 262 ( 0.7) | 134 ( 0.8) |
| 12h_to_2d mortality (%) |  | 2269 ( 1.8) | 619 ( 1.8) | 336 ( 1.9) |
| 12h_to_4d mortality (%) |  | 4119 ( 3.3) | 1135 ( 3.2) | 573 ( 3.3) |
| 12h_to_7d mortality (%) |  | 5936 ( 4.8) | 1609 ( 4.6) | 827 ( 4.7) |
| In-hospital mortality (%) |  | 9993 ( 8.1) | 2791 ( 8.0) | 1440 ( 8.2) |

**Table S2**. Performance of different models on the task of predicting death within the next 24 hours triggered every 4 hours, tested on the MIMIC-IV database.

| Model | Time from Start (hours) | AUROC (%) | 95% CI (%) | AUPRC (%) | 95% CI (%) |
| --- | --- | --- | --- | --- | --- |
| TBAL | 0 | 85 | (81.5, 88.1) | 17.9 | (15.1, 22.6) |
|  | 4 | 88.6 | (86.2, 90.3) | 23.2 | (19.1, 27.6) |
|  | 8 | 93 | (91.7, 94.2) | 31.9 | (27.3, 36.8) |
|  | 12 | 94.2 | (93.4, 95.1) | 36.3 | (31.9, 40.2) |
|  | 16 | 94.4 | (93.3, 95.5) | 37.5 | (32.0, 43.2) |
|  |  |  |  |  |  |
|  | Time, End (hours) | AUROC (%) | 95% CI (%) | AUPRC (%) | 95% CI (%) |
|  | -16 | 95.6 | (95.2, 96.2) | 73.7 | (71.5, 75.8) |
|  | -12 | 97.0 | (96.6, 97.5) | 80.6 | (78.8, 82.1) |
|  | -8 | 97.8 | (97.4, 98.2) | 85.5 | (84.1, 86.9) |
|  | -4 | 98.5 | (98.3, 98.8) | 89.8 | (88.7, 91.1) |
|  | 0 | 98.9 | (98.6, 99.2) | 92.1 | (90.6, 93.7) |
| LSTM | Time from Start (hours) | AUROC (%) | 95% CI (%) | AUPRC (%) | 95% CI (%) |
|  | 0 | 85.5 | (81.7, 89.1) | 20.3 | (14.3, 27.5) |
|  | 4 | 88.9 | (87.0, 90.5) | 24.1 | (20.2, 28.4) |
|  | 8 | 93.3 | (92.2, 94.1) | 32.6 | (28.9, 37.2) |
|  | 12 | 94.3 | (93.3, 95.3) | 38.3 | (34.4, 43.0) |
|  | 16 | 94.4 | (93.6, 95.3) | 40.1 | (35.3, 45.5) |
|  |  |  |  |  |  |
|  | Time, End (hours) | AUROC (%) | 95% CI (%) | AUPRC (%) | 95% CI (%) |
|  | -16 | 96.1 | (95.6, 96.6) | 72.8 | (70.4, 75.1) |
|  | -12 | 97.1 | (96.7, 97.5) | 79.1 | (77.1, 81.3) |
|  | -8 | 97.9 | (97.6, 98.3) | 85.1 | (83.6, 86.6) |
|  | -4 | 98.6 | (98.4, 99.0) | 89.4 | (87.9, 90.8) |
|  | 0 | 99.0 | (98.7, 99.3) | 91.6 | (89.8, 93.0) |

**Table S3.** Performance of different models on the task of predicting death within the next 24 hours triggered every 4 hours, tested on the eICU-CRD database.

| Model | Time from Start (hours) | AUROC (%) | 95% CI (%) | AUPRC (%) | 95% CI (%) |
| --- | --- | --- | --- | --- | --- |
| TBAL | 0 | 88.1 | (86.4, 90.3) | 18.7 | (14.5, 22.5) |
|  | 4 | 89.5 | (87.9, 90.8) | 24.9 | (21.3, 29.4) |
|  | 8 | 91.0 | (90.1, 92.0) | 32.2 | (28.5, 35.2) |
|  | 12 | 91.7 | (90.8, 92.5) | 37.6 | (34.4, 41.3) |
|  | 16 | 92.1 | (91.0, 93.2) | 40.7 | (37.1, 45.2) |
|  |  | | | | |
|  | Time to End (hours) | AUROC (%) | 95% CI (%) | AUPRC (%) | 95% CI (%) |
|  | -16 | 91.3 | (90.6, 91.9) | 62.5 | (60.9, 64.2) |
|  | -12 | 92.3 | (91.7, 92.8) | 65.4 | (63.7, 67.2) |
|  | -8 | 93.4 | (92.7, 94.0) | 68.6 | (67.0, 70.6) |
|  | -4 | 94.7 | (94.0, 95.1) | 72.1 | (69.9, 73.8) |
|  | 0 | 95.4 | (95.0, 95.9) | 74.9 | (73.0, 76.7) |
| LSTM | Time from Start (hours) | AUROC (%) | 95% CI (%) | AUPRC (%) | 95% CI (%) |
|  | 0 | 88.2 | (86.5, 89.9) | 16.8 | (13.4, 19.8) |
|  | 4 | 89.0 | (87.7, 90.2) | 20.5 | (17.8, 23.7) |
|  | 8 | 90.6 | (89.4, 91.6) | 26.9 | (23.9, 30.7) |
|  | 12 | 91.3 | (90.3, 92.1) | 31.5 | (28.0, 35.2) |
|  | 16 | 91.4 | (90.3, 92.4) | 33.1 | (28.8, 35.8) |
|  |  | | | | |
|  | Time to End (hours) | AUROC (%) | 95% CI (%) | AUPRC (%) | 95% CI (%) |
|  | -16 | 90.3 | (89.5, 91.0) | 58.0 | (56.3, 60.0) |
|  | -12 | 91.4 | (90.8, 91.9) | 60.7 | (58.4, 62.3) |
|  | -8 | 92.5 | (92.1, 93.1) | 63.6 | (62.2, 65.5) |
|  | -4 | 94.1 | (93.7, 94.6) | 68.1 | (66.4, 69.8) |
|  | 0 | 94.9 | (94.3, 95.4) | 71.3 | (69.3, 73.8) |

**Table S4**. Summary of the cross-database cross-generalization capability validation of the TBAL model

| Task | Test strategy | AUROC (%) | AUPRC (%) |
| --- | --- | --- | --- |
| In-hospital death prediction triggered at 12th hour after ICU admission | eICU-CRD to eICU-CRD | 86.5 (85.7, 87.6) | 43.5 (41.2, 45.8) |
|  | eICU-CRD to MIMIC-IV | 76.1 (74.6, 77.4) | 34.9 (32.5, 38.1) |
|  | MIMIC-IV to eICU-CRD | 81.3 (80.1, 82.3) | 34.6 (32.7, 37.0) |
|  | MIMIC-IV to MIMIC-IV | 87.4 (86.5, 88.3) | 49.9 (47.5, 52.0) |
| Prediction of death within the next 24 hours, triggered every 4 hours following ICU admission | eICU-CRD to eICU-CRD | 87.0 (86.7, 87.3) | 36.4 (35.9, 37.1) |
|  | eICU-CRD to MIMIC-IV | 83.0 (82.3, 83.7) | 28.3 (27.4, 29.9) |
|  | MIMIC-IV to eICU-CRD | 85.0 (84.6, 85.2) | 43.1 (42.5, 44.0) |
|  | MIMIC-IV to MIMIC-IV | 90.0 (89.6, 90.4) | 41.2 (39.9, 42.7) |

**Table S5.** Variable dictionary in the MIMIC-IV database.

| Index | Variable name^a^ | Value type | Aggregation methods | First-time missing | Subsequent missing | Share |
| --- | --- | --- | --- | --- | --- | --- |
| 1 | temperature | num | mean | nocb | lin | Yes |
| 2 | glucose | num | mean | nocb | lin | Yes |
| 3 | heart_rate | num | mean | nocb | lin | Yes |
| 4 | sbp | num | mean | nocb | lin | Yes |
| 5 | dbp | num | mean | nocb | lin | Yes |
| 6 | mbp | num | mean | nocb | lin |  |
| 7 | resp_rate | num | mean | nocb | lin | Yes |
| 8 | spo2 | num | mean | nocb | lin | Yes |
| 9 | so2 | num | mean | nocb | lin |  |
| 10 | po2 | num | mean | nocb | lin |  |
| 11 | pco2 | num | mean | nocb | lin |  |
| 12 | fio2 | num | mean | nocb | lin | Yes |
| 13 | aado2 | num | mean | nocb | lin |  |
| 14 | aado2_calc | num | mean | nocb | lin |  |
| 15 | pao2fio2ratio | num | mean | nocb | lin |  |
| 16 | ph | num | mean | nocb | lin | Yes |
| 17 | baseexcess | num | mean | nocb | lin | Yes |
| 18 | bicarbonate | num | mean | nocb | lin | Yes |
| 19 | totalco2 | num | mean | nocb | lin | Yes |
| 20 | hematocrit | num | mean | nocb | lin | Yes |
| 21 | hemoglobin | num | mean | nocb | lin | Yes |
| 22 | carboxyhemoglobin | num | mean | nocb | lin |  |
| 23 | methemoglobin | num | mean | nocb | lin |  |
| 24 | chloride | num | mean | nocb | lin | Yes |
| 25 | calcium | num | mean | nocb | lin | Yes |
| 26 | potassium | num | mean | nocb | lin | Yes |
| 27 | lactate | num | mean | nocb | lin | Yes |
| 28 | gcs | ord | median | nocb | locf | Yes |
| 29 | uo | num | sum | cont | zero | Yes |
| 30 | dopamine | num/bin | mean_w/any | zero | zero | Yes |
| 31 | epinephrine | num/bin | mean_w/any | zero | zero | Yes |
| 32 | norepinephrine | num/bin | mean_w/any | zero | zero | Yes |
| 33 | phenylephrine | num/bin | mean_w/any | zero | zero | Yes |
| 34 | vasopressin | num/bin | mean_w/any | zero | zero | Yes |
| 35 | dobutamine | num/bin | mean_w/any | zero | zero | Yes |
| 36 | milrinone | num/bin | mean_w/any | zero | zero | Yes |
| 37 | vent | cat | mode | zero | zero | Yes |
| 38 | mch | num | mean | nocb | lin |  |
| 39 | mchc | num | mean | nocb | lin |  |
| 40 | mcv | num | mean | nocb | lin |  |
| 41 | platelet | num | mean | nocb | lin |  |
| 42 | rbc | num | mean | nocb | lin |  |
| 43 | rdw | num | mean | nocb | lin |  |
| 44 | wbc | num | mean | nocb | lin | Yes |
| 45 | sodium | num | mean | nocb | lin | Yes |
| 46 | albumin | num | mean | nocb | lin | Yes |
| 47 | globulin | num | mean | nocb | lin |  |
| 48 | total_protein | num | mean | nocb | lin |  |
| 49 | aniongap | num | mean | nocb | lin | Yes |
| 50 | bun | num | mean | nocb | lin | Yes |
| 51 | creatinine | num | mean | nocb | lin | Yes |

^a^: These variables are derived from the pipeline defined in this repository: https://github.com/MIT-LCP/mimic-code.

"Share" means that these variables have the same concepts in both MIMIC-IV and eICU-CRD.

**Table S6**. Variable dictionary in the eICU-CRD database.

| Index | Variable name^a^ | Value type | Aggregation methods | First-time missing | Subsequent missing | Share |
| --- | --- | --- | --- | --- | --- | --- |
| 1 | fio2 | num | mean | nocb | lin | Yes |
| 2 | peep | num | mean | nocb | lin |  |
| 3 | pao2 | num | mean | nocb | lin |  |
| 4 | ph | num | mean | nocb | lin | Yes |
| 5 | baseexcess | num | mean | nocb | lin | Yes |
| 6 | aniongap | num | mean | nocb | lin | Yes |
| 7 | temperature | num | mean | nocb | lin | Yes |
| 8 | nibp_systolic | num | mean | nocb | lin | Yes |
| 9 | nibp_diastolic | num | mean | nocb | lin | Yes |
| 10 | respiratoryrate | num | mean | nocb | lin | Yes |
| 11 | spo2 | num | mean | nocb | lin | Yes |
| 12 | gcs | ord | median | nocb | locf | Yes |
| 13 | uo | num | sum | cont | zero | Yes |
| 14 | dopamine | bin | any | zero | zero | Yes |
| 15 | epinephrine | bin | any | zero | zero | Yes |
| 16 | norepinephrine | bin | any | zero | zero | Yes |
| 17 | phenylephrine | bin | any | zero | zero | Yes |
| 18 | vasopressin | bin | any | zero | zero | Yes |
| 19 | dobutamine | bin | any | zero | zero | Yes |
| 20 | milrinone | bin | any | zero | zero | Yes |
| 21 | heparin | bin | any | zero | zero |  |
| 22 | warfarin | bin | any | zero | zero |  |
| 23 | o2 | num | mean | nocb | locf | Yes |
| 24 | bicarbonate | num | mean | nocb | lin | Yes |
| 25 | totalco2 | num | mean | nocb | lin | Yes |
| 26 | hematocrit | num | mean | nocb | lin | Yes |
| 27 | hemoglobin | num | mean | nocb | lin | Yes |
| 28 | chloride | num | mean | nocb | lin | Yes |
| 29 | calcium | num | mean | nocb | lin | Yes |
| 30 | potassium | num | mean | nocb | lin | Yes |
| 31 | sodium | num | mean | nocb | lin | Yes |
| 32 | lactate | num | mean | nocb | lin | Yes |
| 33 | glucose | num | mean | nocb | lin | Yes |
| 34 | albumin | num | mean | nocb | lin | Yes |
| 35 | bun | num | mean | nocb | lin | Yes |
| 36 | creatinine | num | mean | nocb | lin | Yes |
| 37 | wbc | num | mean | nocb | lin | Yes |
| 38 | bands | num | mean | nocb | lin |  |
| 39 | alt | num | mean | nocb | lin |  |
| 40 | ast | num | mean | nocb | lin |  |
| 41 | alp | num | mean | nocb | lin |  |
| 42 | heart_rate | num | mean | nocb | lin | Yes |

^a^: These variables are derived from the pipeline defined in this repository: https://github.com/MIT-LCP/eicu-code.

"Share" means that these variables have the same concepts in both MIMIC-IV and eICU-CRD.

**Table S7**. Summary of supplementary performance for static tasks in the MIMIC-IV database

| Tasks | model | Accuracy | Recall | Precision | F1 |
| --- | --- | --- | --- | --- | --- |
| 12 h to 1 d mortality | TBAL | 98.9 (98.4, 99.2) | 50.8 (38.6, 63.2) | 50.0 (38.2, 72.8) | 50.4 (46.2, 58.6) |
|  | LSTM | 98.7 (98.0, 99.0) | 40.6 (33.2, 60.2) | 41.3 (28.0, 51.4) | 40.9 (35.1, 46.4) |
| 12 h to 2 d mortality | TBAL | 97.8 (96.9, 98.2) | 45.2 (39.0, 59.8) | 48.7 (37.6, 58.1) | 46.9 (42.9, 51.1) |
|  | LSTM | 97.3 (97.1, 97.8) | 51.2 (42.0, 57.1) | 41.4 (38.7, 53.0) | 45.8 (43.2, 50.9) |
| 12 h to 4 d mortality | TBAL | 95.7 (95.2, 96.7) | 50.9 (40.4, 55.9) | 44.6 (40.5, 59.4) | 47.6 (43.6, 51.7) |
|  | LSTM | 95.4 (94.8, 96.1) | 52.8 (46.4, 59.4) | 41.9 (37.6, 48.3) | 46.7 (43.6, 49.8) |
| 12 h to 7 d mortality | TBAL | 93.2 (92.5, 93.7) | 53.2 (49.7, 58.0) | 40.9 (38.1, 44.1) | 46.2 (44.2, 49.2) |
|  | LSTM | 93.0 (91.1, 94.2) | 50.6 (43.4, 63.7) | 39.5 (32.0, 44.4) | 44.4 (41.8, 46.5) |
| In-hospital mortality | TBAL | 89.3 (87.7, 90.5) | 58.8 (53.1, 67.3) | 46.7 (41.9, 51.8) | 52.1 (50.2, 54.8) |
|  | LSTM | 88.8 (86.7, 89.7) | 58.6 (54.9, 67.7) | 44.9 (40.2, 49.0) | 50.9 (48.7, 53.9) |
| ICU LoS > 2d | TBAL | 82.8 (82.2, 83.6) | 99.9 (98.4, 100.0) | 82.8 (82.2, 84.0) | 90.5 (90.2, 91.0) |
|  | LSTM | 82.6 (82.3, 83.3) | 99.9 (99.4, 100.0) | 82.7 (82.4, 83.5) | 90.5 (90.3, 90.9) |

**Table S8**. Summary of supplementary performance for static tasks in the eICU-CRD database

| Tasks | model | Accuracy | Recall | Precision | F1 |
| --- | --- | --- | --- | --- | --- |
| 12 h to 1 d mortality | TBAL | 99.0 (98.9, 99.4) | 41.0 (22.0, 46.2) | 26.4 (22.2, 43.8) | 32.2 (26.5, 36.8) |
|  | LSTM | 99.1 (98.7, 99.4) | 27.6 (19.9, 43.8) | 26.1 (16.2, 39.3) | 26.8 (22.5, 32.9) |
| 12 h to 2 d mortality | TBAL | 97.6 (97.4, 98.4) | 45.8 (30.5, 49.6) | 32.1 (27.1, 46.1) | 37.7 (32.5, 41.0) |
|  | LSTM | 97.9 (96.7, 98.2) | 32.1 (29.3, 49.1) | 33.4 (23.9, 39.9) | 32.7 (29.3, 37.5) |
| 12 h to 4 d mortality | TBAL | 96.5 (95.9, 96.8) | 42.2 (39.0, 48.1) | 41.0 (35.3, 45.2) | 41.6 (38.7, 45.0) |
|  | LSTM | 95.8 (95.5, 96.2) | 46.2 (42.9, 49.5) | 34.8 (32.9, 37.8) | 39.7 (37.3, 42.5) |
| 12 h to 7 d mortality | TBAL | 94.8 (94.2, 95.6) | 47.7 (41.6, 51.7) | 39.3 (35.7, 45.6) | 43.1 (41.4, 45.4) |
|  | LSTM | 94.3 (93.8, 95.6) | 48.0 (39.4, 52.8) | 36.2 (31.6, 44.2) | 41.3 (38.3, 43.6) |
| In-hospital mortality | TBAL | 91.4 (90.4, 91.6) | 52.6 (51.2, 59.1) | 41.3 (37.4, 42.6) | 46.3 (44.4, 48.2) |
|  | LSTM | 91.2 (90.0, 92.4) | 51.3 (45.5, 57.7) | 40.4 (36.5, 47.1) | 45.2 (43.8, 47.9) |
| ICU LoS > 2d | TBAL | 79.8 (79.3, 80.4) | 100.0 (99.7, 100.0) | 79.8 (79.4, 80.5) | 88.8 (88.5, 89.1) |
|  | LSTM | 79.8 (79.4, 80.3) | 100.0 (100.0, 100.0) | 79.8 (79.4, 80.3) | 88.8 (88.5, 89.1) |

**Table S9**. Hyperparameter settings for the development of the TBAL and LSTM models

| Hyperparameter | TBAL Model | LSTM Baseline Model |
| --- | --- | --- |
| Hidden size | 512 | 512 |
| Number of LSTM layers | 1 | 1 |
| Attention mechanism | Yes | No |
| Batch size | 300 | 300 |
| Learning rate | 0.001 | 0.001 |
| Optimizer | Adam | Adam |
| Number of epochs | 100 | 100 |
| Dropout rate | 0.2 | 0.2 |
| L2 regularization weight | 0.001 | 0.001 |
| Gradient clipping | No | No |
| Early stopping | Yes | Yes |
| Time-aware encoding | Yes | No |
| Mask vector input | Yes | No |

**Table S10**. Top 20 feature importance rankings for the static task across different age subgroups in MIMIC-IV

| <65 | | >=65 | |
| --- | --- | --- | --- |
| Feature | IG | Feature | IG |
| UO | 0.19 | UO | 0.22 |
| BUN | 0.17 | BUN | 0.18 |
| Resp_rate | 0.13 | Resp_rate | 0.14 |
| Albumin | 0.13 | Albumin | 0.14 |
| GCS (mask) | 0.11 | Temperature | 0.12 |
| Temperature | 0.11 | GCS (mask) | 0.12 |
| Creatinine | 0.10 | Glucose (mask) | 0.11 |
| Lactate | 0.10 | Creatinine | 0.11 |
| Fio2_chartevents | 0.09 | Lactate | 0.11 |
| Glucose (mask) | 0.09 | Fio2_chartevents | 0.10 |
| UO (mask) | 0.07 | UO (mask) | 0.08 |
| GCS | 0.06 | GCS | 0.08 |
| SBP | 0.06 | SBP | 0.07 |
| Aniongap | 0.06 | WBC | 0.06 |
| DT | 0.05 | Aniongap | 0.06 |
| WBC | 0.05 | DT | 0.06 |
| SBP (mask) | 0.05 | DBP (mask) | 0.06 |
| DBP (mask) | 0.05 | SBP (mask) | 0.06 |
| Totalco2 | 0.05 | Totalco2 | 0.05 |
| Bicarbonate | 0.05 | Bicarbonate | 0.05 |

**Table S11**. Top 20 feature importance rankings for the static task across different race subgroups in MIMIC-IV

| Asian | | Black/African American | | Hispanic/Latino | | Other/Unknown | | White | |
| --- | --- | --- | --- | --- | --- | --- | --- | --- | --- |
| Feature | IG | Feature | IG | Feature | IG | Feature | IG | Feature | IG |
| UO | 0.16 | UO | 0.21 | UO | 0.19 | UO | 0.22 | UO | 0.22 |
| BUN | 0.14 | BUN | 0.17 | BUN | 0.16 | BUN | 0.20 | BUN | 0.19 |
| Albumin | 0.11 | Resp_rate | 0.13 | Albumin | 0.12 | GCS (mask) | 0.15 | Resp_rate | 0.14 |
| Resp_rate | 0.11 | Albumin | 0.13 | Resp_rate | 0.12 | Resp_rate | 0.15 | Albumin | 0.14 |
| GCS (mask) | 0.09 | Temperature | 0.11 | GCS (mask) | 0.12 | Albumin | 0.15 | Temperature | 0.12 |
| Temperature | 0.09 | Creatinine | 0.11 | Temperature | 0.10 | Temperature | 0.11 | GCS (mask) | 0.11 |
| Creatinine | 0.08 | Lactate | 0.10 | Creatinine | 0.10 | Creatinine | 0.11 | Creatinine | 0.11 |
| Lactate | 0.08 | Fio2_chartevents | 0.10 | Lactate | 0.10 | Lactate | 0.11 | Lactate | 0.11 |
| Fio2_chartevents | 0.08 | GCS (mask) | 0.09 | Glucose (mask) | 0.09 | Glucose (mask) | 0.10 | Glucose (mask) | 0.11 |
| Glucose (mask) | 0.08 | Glucose (mask) | 0.09 | Fio2_chartevents | 0.09 | Fio2_chartevents | 0.10 | Fio2_chartevents | 0.10 |
| UO (mask) | 0.07 | GCS | 0.07 | UO (mask) | 0.08 | UO (mask) | 0.10 | UO (mask) | 0.08 |
| GCS | 0.05 | SBP | 0.07 | GCS | 0.06 | GCS | 0.07 | GCS | 0.07 |
| SBP | 0.05 | UO (mask) | 0.07 | WBC | 0.06 | SBP | 0.06 | SBP | 0.06 |
| SBP (mask) | 0.05 | SBP (mask) | 0.06 | DT | 0.05 | Aniongap | 0.06 | WBC | 0.06 |
| DBP (mask) | 0.05 | DBP (mask) | 0.06 | Aniongap | 0.05 | DBP (mask) | 0.06 | Aniongap | 0.06 |
| Aniongap | 0.05 | WBC | 0.06 | SBP | 0.05 | WBC | 0.06 | DT | 0.06 |
| DT | 0.04 | Aniongap | 0.06 | SBP (mask) | 0.05 | SBP (mask) | 0.06 | SBP (mask) | 0.06 |
| WBC | 0.04 | DT | 0.05 | DBP (mask) | 0.05 | DT | 0.06 | DBP (mask) | 0.06 |
| Totalco2 | 0.04 | Totalco2 | 0.05 | Totalco2 | 0.05 | Totalco2 | 0.06 | Totalco2 | 0.05 |
| Bicarbonate | 0.04 | Spo2 (mask) | 0.05 | Bicarbonate | 0.05 | Bicarbonate | 0.06 | Bicarbonate | 0.05 |

**Table S12**. Top 20 feature importance rankings for the dynamic task across different age subgroups in MIMIC-IV

| <65 | | >=65 | |
| --- | --- | --- | --- |
| Spo2 (mask) | 0.10 | Spo2 (mask) | 0.12 |
| SBP (mask) | 0.10 | SBP (mask) | 0.12 |
| Lactate | 0.09 | Lactate | 0.11 |
| DBP (mask) | 0.09 | DBP (mask) | 0.10 |
| UO | 0.08 | UO | 0.10 |
| DBP | 0.08 | DBP | 0.10 |
| Spo2 | 0.07 | Spo2 | 0.09 |
| GCS | 0.06 | GCS | 0.08 |
| WBC | 0.06 | WBC | 0.07 |
| PH | 0.06 | PH | 0.07 |
| UO (mask) | 0.05 | UO (mask) | 0.06 |
| BUN | 0.04 | BUN | 0.05 |
| Aniongap | 0.04 | Sodium | 0.05 |
| Resp_rate (mask) | 0.04 | Aniongap | 0.05 |
| Creatinine | 0.04 | Resp_rate (mask) | 0.05 |
| Sodium | 0.04 | Creatinine | 0.05 |
| Fio2_chartevents | 0.04 | Fio2_chartevents | 0.04 |
| Resp_rate | 0.03 | SBP | 0.04 |
| SBP | 0.03 | Resp_rate | 0.04 |
| Glucose (mask) | 0.03 | Glucose (mask) | 0.03 |
| Spo2 (mask) | 0.10 | Spo2 (mask) | 0.12 |

**Table S13**. Top 20 feature importance rankings for the dynamic task across different race subgroups in MIMIC-IV

| Asian | | Black/African American | | Hispanic/Latino | | Other/Unknown | | White | |
| --- | --- | --- | --- | --- | --- | --- | --- | --- | --- |
| Feature | IG | Feature | IG | Feature | IG | Feature | IG | Feature | IG |
| Spo2 (mask) | 0.10 | Spo2 (mask) | 0.12 | Spo2 (mask) | 0.13 | Spo2 (mask) | 0.12 | Spo2 (mask) | 0.12 |
| SBP (mask) | 0.09 | SBP (mask) | 0.12 | SBP (mask) | 0.13 | SBP (mask) | 0.11 | SBP (mask) | 0.12 |
| Lactate | 0.09 | Lactate | 0.11 | Lactate | 0.12 | Lactate | 0.10 | Lactate | 0.11 |
| DBP (mask) | 0.08 | DBP (mask) | 0.10 | DBP (mask) | 0.11 | DBP (mask) | 0.10 | DBP (mask) | 0.10 |
| DBP | 0.07 | UO | 0.10 | DBP | 0.10 | DBP | 0.08 | UO | 0.09 |
| UO | 0.06 | DBP | 0.10 | UO | 0.10 | UO | 0.08 | DBP | 0.09 |
| Spo2 | 0.06 | Spo2 | 0.08 | Spo2 | 0.09 | Spo2 | 0.07 | Spo2 | 0.08 |
| UO (mask) | 0.06 | GCS | 0.08 | GCS | 0.08 | Sodium | 0.07 | GCS | 0.08 |
| Sodium | 0.06 | WBC | 0.07 | WBC | 0.08 | WBC | 0.07 | WBC | 0.07 |
| WBC | 0.05 | PH | 0.07 | PH | 0.07 | GCS | 0.07 | PH | 0.07 |
| GCS | 0.05 | UO (mask) | 0.06 | Sodium | 0.06 | PH | 0.06 | UO (mask) | 0.06 |
| PH | 0.05 | BUN | 0.05 | BUN | 0.06 | UO (mask) | 0.06 | Resp_rate (mask) | 0.05 |
| Resp_rate (mask) | 0.04 | Resp_rate (mask) | 0.05 | Resp_rate (mask) | 0.06 | Resp_rate (mask) | 0.05 | Aniongap | 0.05 |
| Creatinine | 0.04 | Aniongap | 0.05 | Aniongap | 0.06 | BUN | 0.05 | Sodium | 0.05 |
| Aniongap | 0.04 | Creatinine | 0.05 | Creatinine | 0.05 | Creatinine | 0.05 | BUN | 0.05 |
| BUN | 0.03 | Sodium | 0.05 | Fio2_chartevents | 0.05 | Aniongap | 0.05 | Creatinine | 0.05 |
| Fio2_chartevents | 0.03 | Fio2_chartevents | 0.05 | UO (mask) | 0.05 | Fio2_chartevents | 0.04 | Fio2_chartevents | 0.04 |
| Resp_rate | 0.03 | SBP | 0.04 | Norepinephrine (mask) | 0.04 | SBP | 0.04 | Resp_rate | 0.04 |
| Vent | 0.03 | Resp_rate | 0.04 | SBP | 0.04 | Resp_rate | 0.03 | SBP | 0.03 |
| SBP | 0.02 | Norepinephrine (mask) | 0.04 | Norepinephrine | 0.04 | Vent | 0.03 | Vent | 0.03 |

**Table S14**. Top 20 feature importance rankings for the static task across different age subgroups in eICU-CRD

| <65 | | >=65 | |
| --- | --- | --- | --- |
| Feature | IG | Feature | IG |
| Lactate | 0.13 | Lactate | 0.16 |
| GCS | 0.12 | GCS | 0.16 |
| BUN | 0.11 | BUN | 0.13 |
| Resp_rate | 0.09 | Resp_rate | 0.11 |
| Chloride | 0.08 | Chloride | 0.11 |
| UO | 0.08 | UO | 0.10 |
| Fio2_chartevents | 0.08 | Spo2 | 0.09 |
| Spo2 | 0.07 | Fio2_chartevents | 0.09 |
| Temperature | 0.07 | Temperature | 0.08 |
| Albumin | 0.06 | Albumin | 0.08 |
| SBP | 0.05 | SBP | 0.06 |
| Hemoglobin | 0.04 | Hemoglobin | 0.05 |
| Creatinine | 0.04 | Creatinine | 0.05 |
| Sodium | 0.04 | Sodium | 0.05 |
| Glucose (mask) | 0.03 | Glucose (mask) | 0.04 |
| WBC | 0.03 | WBC | 0.04 |
| Norepinephrine (mask) | 0.03 | Norepinephrine (mask) | 0.03 |
| Phenylephrine (mask) | 0.02 | Baseexcess | 0.02 |
| Vasopressin (mask) | 0.02 | Vent (mask) | 0.02 |
| Baseexcess | 0.02 | Phenylephrine (mask) | 0.02 |

**Table S15**. Top 20 feature importance rankings for the static task across different race subgroups in eICU-CRD

| Asian | | Black/African American | | Hispanic/Latino | | Other/Unknown | | White | |
| --- | --- | --- | --- | --- | --- | --- | --- | --- | --- |
| Feature | IG | Feature | IG | Feature | IG | Feature | IG | Feature | IG |
| GCS | 0.14 | Lactate | 0.11 | Lactate | 0.11 | Lactate | 0.16 | Lactate | 0.12 |
| Lactate | 0.13 | GCS | 0.11 | GCS | 0.11 | GCS | 0.14 | GCS | 0.12 |
| BUN | 0.11 | BUN | 0.09 | BUN | 0.09 | BUN | 0.13 | BUN | 0.10 |
| Resp_rate | 0.09 | Resp_rate | 0.08 | Resp_rate | 0.08 | Resp_rate | 0.11 | Resp_rate | 0.08 |
| Chloride | 0.09 | Chloride | 0.07 | Chloride | 0.07 | Chloride | 0.10 | Chloride | 0.08 |
| UO | 0.08 | UO | 0.07 | UO | 0.07 | UO | 0.10 | UO | 0.07 |
| Fio2_chartevents | 0.08 | Fio2_chartevents | 0.06 | Spo2 | 0.06 | Spo2 | 0.09 | Spo2 | 0.07 |
| Spo2 | 0.07 | Spo2 | 0.06 | Fio2_chartevents | 0.06 | Fio2_chartevents | 0.09 | Fio2_chartevents | 0.07 |
| Albumin | 0.06 | Temperature | 0.05 | Temperature | 0.06 | Temperature | 0.08 | Temperature | 0.06 |
| Temperature | 0.06 | Albumin | 0.05 | Albumin | 0.05 | Albumin | 0.08 | Albumin | 0.06 |
| SBP | 0.05 | Hemoglobin | 0.04 | Hemoglobin | 0.04 | SBP | 0.06 | Glucose (mask) | 0.04 |
| Glucose (mask) | 0.05 | SBP | 0.04 | SBP | 0.04 | Hemoglobin | 0.06 | SBP | 0.04 |
| Hemoglobin | 0.04 | Sodium | 0.03 | Sodium | 0.03 | Creatinine | 0.05 | Hemoglobin | 0.04 |
| Sodium | 0.04 | Creatinine | 0.03 | Creatinine | 0.03 | Sodium | 0.04 | Sodium | 0.04 |
| Creatinine | 0.04 | WBC | 0.03 | Glucose (mask) | 0.03 | Glucose (mask) | 0.04 | Creatinine | 0.03 |
| WBC | 0.03 | Norepinephrine (mask) | 0.02 | WBC | 0.03 | Norepinephrine (mask) | 0.04 | WBC | 0.03 |
| Norepinephrine (mask) | 0.03 | Glucose (mask) | 0.02 | Vent (mask) | 0.03 | WBC | 0.03 | Norepinephrine (mask) | 0.03 |
| Baseexcess | 0.02 | Phenylephrine (mask) | 0.02 | Norepinephrine (mask) | 0.02 | Phenylephrine (mask) | 0.02 | Phenylephrine (mask) | 0.02 |
| Phenylephrine (mask) | 0.02 | Vent (mask) | 0.02 | Baseexcess | 0.02 | Vasopressin (mask) | 0.02 | Vasopressin (mask) | 0.02 |
| Vasopressin (mask) | 0.02 | Vasopressin (mask) | 0.02 | Phenylephrine (mask) | 0.01 | Baseexcess | 0.02 | Spo2 (mask) | 0.02 |

**Table S16**. Top 20 feature importance rankings for the dynamic task across different age subgroups in eICU-CRD

| <65 | | >=65 | |
| --- | --- | --- | --- |
| GCS | 0.24 | GCS | 0.22 |
| SBP | 0.17 | SBP | 0.15 |
| Lactate | 0.14 | Lactate | 0.13 |
| UO | 0.13 | UO | 0.12 |
| Baseexcess | 0.13 | Baseexcess | 0.12 |
| Spo2 | 0.12 | Spo2 | 0.11 |
| Potassium | 0.11 | Potassium | 0.10 |
| WBC | 0.11 | WBC | 0.10 |
| Resp_rate (mask) | 0.09 | BUN | 0.08 |
| Aniongap | 0.08 | PH | 0.08 |
| BUN | 0.08 | Aniongap | 0.07 |
| PH | 0.08 | DBP | 0.06 |
| DBP | 0.07 | Resp_rate (mask) | 0.06 |
| Resp_rate | 0.07 | Temperature | 0.06 |
| Temperature | 0.07 | Sodium | 0.06 |
| Spo2 (mask) | 0.07 | Resp_rate | 0.06 |
| DBP (mask) | 0.06 | Spo2 (mask) | 0.05 |
| Vasopressin (mask) | 0.06 | Bicarbonate | 0.05 |
| Sodium | 0.06 | Vasopressin (mask) | 0.05 |
| Epinephrine (mask) | 0.06 | Epinephrine (mask) | 0.05 |
| GCS | 0.24 | GCS | 0.22 |

**Table S17**. Top 20 feature importance rankings for the dynamic task across different race subgroups in eICU-CRD

| Asian | | Black/African American | | Hispanic/Latino | | Other/Unknown | | White | |
| --- | --- | --- | --- | --- | --- | --- | --- | --- | --- |
| Feature | IG | Feature | IG | Feature | IG | Feature | IG | Feature | IG |
| GCS | 0.26 | GCS | 0.24 | GCS | 0.24 | GCS | 0.24 | GCS | 0.23 |
| SBP | 0.18 | SBP | 0.17 | SBP | 0.16 | SBP | 0.17 | SBP | 0.16 |
| Lactate | 0.15 | Lactate | 0.14 | Lactate | 0.13 | Lactate | 0.14 | Lactate | 0.13 |
| UO | 0.14 | UO | 0.13 | UO | 0.13 | Baseexcess | 0.13 | Baseexcess | 0.12 |
| Baseexcess | 0.14 | Baseexcess | 0.13 | Baseexcess | 0.13 | UO | 0.13 | UO | 0.12 |
| Spo2 | 0.13 | Spo2 | 0.12 | Spo2 | 0.12 | Spo2 | 0.12 | Spo2 | 0.11 |
| Potassium | 0.12 | Potassium | 0.11 | WBC | 0.11 | Potassium | 0.11 | Potassium | 0.11 |
| WBC | 0.12 | WBC | 0.11 | Potassium | 0.11 | WBC | 0.11 | WBC | 0.10 |
| PH | 0.09 | Aniongap | 0.08 | DBP (mask) | 0.08 | BUN | 0.08 | BUN | 0.08 |
| BUN | 0.09 | PH | 0.08 | BUN | 0.08 | PH | 0.08 | PH | 0.08 |
| Aniongap | 0.09 | BUN | 0.08 | PH | 0.08 | Aniongap | 0.08 | Aniongap | 0.08 |
| DBP | 0.08 | Vasopressin (mask) | 0.07 | Aniongap | 0.08 | DBP | 0.07 | DBP | 0.07 |
| Temperature | 0.07 | DBP | 0.07 | DBP | 0.07 | Resp_rate (mask) | 0.07 | Vasopressin (mask) | 0.07 |
| Resp_rate | 0.07 | Epinephrine (mask) | 0.07 | SBP (mask) | 0.07 | Resp_rate | 0.07 | Resp_rate | 0.07 |
| Sodium | 0.07 | Temperature | 0.07 | Temperature | 0.07 | Temperature | 0.06 | Temperature | 0.06 |
| Vasopressin (mask) | 0.06 | Resp_rate | 0.07 | Sodium | 0.06 | DBP (mask) | 0.06 | Epinephrine (mask) | 0.06 |
| Bicarbonate | 0.06 | Sodium | 0.06 | Resp_rate (mask) | 0.06 | Sodium | 0.06 | Resp_rate (mask) | 0.06 |
| Epinephrine (mask) | 0.06 | Resp_rate (mask) | 0.06 | Spo2 (mask) | 0.06 | Bicarbonate | 0.05 | Sodium | 0.06 |
| Hematocrit | 0.06 | Bicarbonate | 0.06 | Resp_rate | 0.06 | Hematocrit | 0.05 | Bicarbonate | 0.05 |
| Chloride | 0.05 | Hematocrit | 0.05 | Bicarbonate | 0.05 | SBP (mask) | 0.05 | Spo2 (mask) | 0.05 |
